# Comparative associations of oximetry patterns in Obstructive Sleep Apnea with incident cardiovascular disease

**DOI:** 10.1093/sleep/zsac179

**Published:** 2022-07-27

**Authors:** Kate Sutherland, Nadi Sadr, Yu Sun Bin, Kristina Cook, Hasthi U Dissanayake, Peter A Cistulli, Philip de Chazal

**Affiliations:** Sleep Research Group, Charles Perkins Centre, University of Sydney, Sydney, New South Wales, Australia; Northern Clinical School, Faculty of Medicine and Health, University of Sydney, Sydney, New South Wales, Australia; Sleep Research Group, Charles Perkins Centre, University of Sydney, Sydney, New South Wales, Australia; Department of Biomedical Informatics, Emory University, Atlanta, Georgia, USA; Sleep Research Group, Charles Perkins Centre, University of Sydney, Sydney, New South Wales, Australia; Northern Clinical School, Faculty of Medicine and Health, University of Sydney, Sydney, New South Wales, Australia; Sleep Research Group, Charles Perkins Centre, University of Sydney, Sydney, New South Wales, Australia; Northern Clinical School, Faculty of Medicine and Health, University of Sydney, Sydney, New South Wales, Australia; Sleep Research Group, Charles Perkins Centre, University of Sydney, Sydney, New South Wales, Australia; Northern Clinical School, Faculty of Medicine and Health, University of Sydney, Sydney, New South Wales, Australia; Sleep Research Group, Charles Perkins Centre, University of Sydney, Sydney, New South Wales, Australia; Northern Clinical School, Faculty of Medicine and Health, University of Sydney, Sydney, New South Wales, Australia; Department of Respiratory and Sleep Medicine, Royal North Shore Hospital, St Leonards, New South Wales, Australia; Sleep Research Group, Charles Perkins Centre, University of Sydney, Sydney, New South Wales, Australia; Northern Clinical School, Faculty of Medicine and Health, University of Sydney, Sydney, New South Wales, Australia; School of Biomedical Engineering, University of Sydney, Sydney, New South Wales, Australia

**Keywords:** Obstructive Sleep Apnea, cardiovascular disease, pulse oximetry, hypoxia, phenotyping

## Abstract

**Study Objectives:**

Intermittent hypoxia is a key mechanism linking Obstructive Sleep Apnea (OSA) to cardiovascular disease (CVD). Oximetry analysis could enhance understanding of which OSA phenotypes are associated with CVD risk. The aim of this study was to compare associations of different oximetry patterns with incident CVD in men and women with OSA.

**Methods:**

Sleep Heart Health Study data were used for analysis. *n* = 2878 Participants (51.8% female; mean age 63.5 ± 10.5 years) with OSA (Apnea Hypopnea Index [AHI] ≥ 5 events/h) and no pre-existing CVD at baseline or within the first 2 years of follow-up were included. Four oximetry analysis approaches were applied: desaturation characteristics, time series analysis, power spectral density, and non-linear analysis. Thirty-one resulting oximetry patterns were compared to incident CVD using proportional hazards regression models adjusted for age, race, smoking, BMI, and sex.

**Results:**

There were no associations between OSA oximetry patterns and incident CVD in the total sample or in men. In women, there were some associations between incident CVD and time series analysis (e.g. SpO_2_ distribution standard deviation, HR 0.81, 95% CI 0.68–0.96, *p* = 0.014) and power spectral density oximetry patterns (e.g. Full frequency band mean HR 0.75; 95% CI 0.59–0.95; *p* = 0.015).

**Conclusions:**

Comprehensive comparison of baseline oximetry patterns in OSA found none were related to development of CVD. There were no standout individual oximetry patterns that appear to be candidates for CVD risk phenotyping in OSA, but some showed marginal relationships with CVD risk in women. Further work is required to understand whether OSA phenotypes can be used to predict susceptibility to cardiovascular disease.

**Statement of Significance**

Intermittent hypoxia resulting from OSA contributes to cardiovascular disease (CVD). Oximetry analysis could be a simple method to phenotype and differentiate those with OSA at most risk of CVD. We compare a range of oximetry patterns from different analytic approaches for association with incident CVD. Within an OSA sample without known CVD at baseline, we did not find evidence for discriminatory ability of any of a comprehensive suite of oximetry patterns with future CVD. Future work to look at relationship to specific CVD types and in combination with other types of signal biomarkers is warranted for clinical OSA phenotyping.

## Introduction

Obstructive Sleep Apnea (OSA) is a common sleep disorder characterized by repetitive collapse of the pharyngeal airway which inhibits airflow. The immediate consequences of obstructed breathing include exaggerated intra-thoracic pressure swings, intermittent hypoxia, and cortical arousal from sleep. These perturbations initiate downstream consequences such as sympathetic activation, inflammation, oxidative stress, cardiac remodeling, and metabolic dysregulation which over time increase cardiovascular risk [1]. OSA is an independent risk factor for a number of cardiovascular diseases (CVD) [2–5].

However, OSA does not lead to CVD in everyone and the standard clinical measure of OSA severity, the Apnea Hypopnea Index (AHI), is a relatively modest indicator of future CVD [6]. AHI indicates the number of airway obstructions per hour of sleep and has been critiqued for not capturing the systemic consequences of OSA, which may more closely link to cardiovascular comorbidity [6–8]. There is scope for enhanced phenotyping of polysomnographic signals to ascertain likelihood of specific OSA consequences, such as CVD [7].

Intermittent hypoxia is a key perturbation which links OSA to CVD pathophysiology [1]. Pulse oximetry is ubiquitous in polysomnography and is used to assist the definition of hypopnea events [9]. Conventionally reported oximetry parameters, such as oxygen desaturation index (ODI), time below 90% saturation (T90), and minimum oxygen saturation, relate to all-cause, and cardiovascular mortality [10]. Recent work to incorporate oxygen desaturation into OSA severity measures show closer relationship with CVD risk factors [11, 12] and mortality [13, 14] than AHI. This additional oximetry information includes length, depth, or area of respiratory-related desaturation events [12–16]. In addition to desaturation characteristics, there is potential for other aspects of the oximetry signal to have utility in OSA phenotyping for CVD risk.

**Figure 1.**
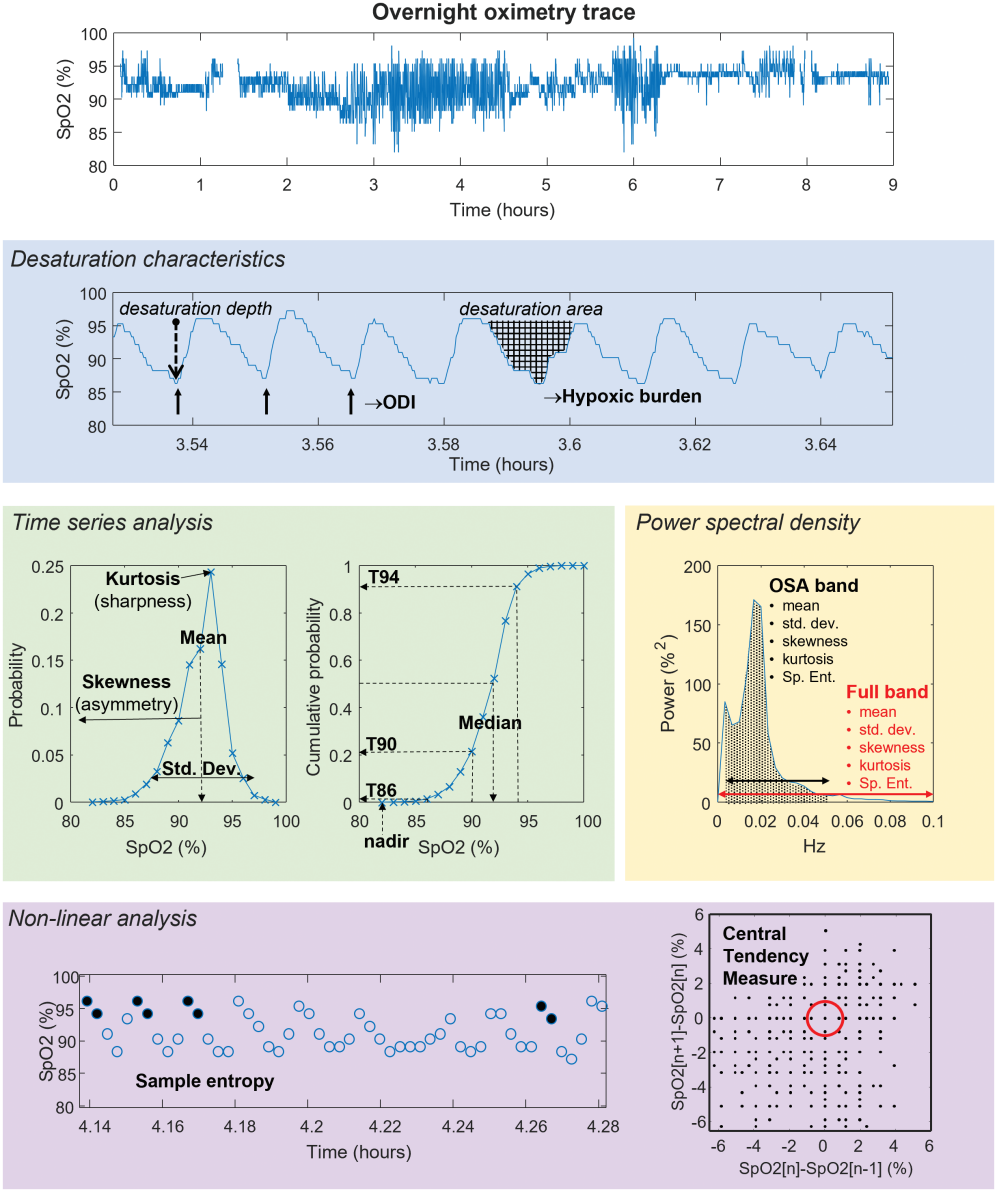
Graphical representation of the oximetry patterns, by four analysis methodologies, assessed in this study. The full night pulse oximetry (SpO_2_) trace is shown in a single participant in the Sleep Heart Health study. The oximetry patterns are grouped by the four analysis types. *Desaturation characteristics*: respiratory events can lead to associated oxygen desaturation events that were measured as a rate of events at fixed thresholds (Oxygen Desaturation Index, ODI) and as a total area under the SpO_2_ curve per hour of sleep (hypoxic burden). For *time series analysis*, the shape of the distribution of the SpO_2_ values was determined by its first four moments (mean, variance, skewness, and kurtosis values). The T80–T94, median, and nadir were determined from the cumulative distribution of the SpO_2_ values. For *power spectral density* analysis, the first four moments and the spectral entropy were calculated from the full-band PSD curve and the OSA-band portion of the curve. For *non-linear analysis*, the sample entropy, and central tendency measure were calculated. Sample entropy measures the regularity of occurrence of similar patterns in the SpO_2_ signal. The central tendency measure was determined from a Poincaré plot of the first difference of the oximetry signal. It is calculated as the proportion of points that fall with the region denoted by the red circle to the total number of points in the plot. The signals used for these graphical representations were taken from participant 245 in the Sleep Heart Health Study database (shhs1-200245.edf).

Oximetry analysis can be performed using different analytic approaches. A convenient framework is to group into four analytic types (illustrated in Figure 1): (1) *desaturation characteristics* (depth, duration, area), (2) *time series analysis* (distribution of saturation values), (3) *power spectral density* (frequency representation of oximetry signal), and (4) *non-linear analysis* (regularity of oximetry patterns) [17]. To our knowledge, a systematic comparison of oximetry patterns in people with OSA, inclusive of these multiple analytic approaches, has not been carried out, and could provide important insights for cardiovascular phenotyping in the sleep clinic [18–20]. If OSA oximetry patterns can differentiate those who go on to develop CVD, this provides a potential phenotyping tool to identify patients with increased CVD risk following OSA diagnosis. The aim of this study was to compare baseline oximetry patterns in those with OSA on the strength of their relationships with future CVD. The overarching goal is to provide information on which oximetry patterns within OSA have potential for predicting CVD risk towards a tool for OSA phenotyping in sleep clinic populations.

## Methods

### Study sample

Data from the Sleep Heart Health Study (SHHS), an open-access dataset hosted by the National Sleep Research Resource was used [21, 22]. The SHHS has been previously described [23]. Inclusion criteria for this analysis were presence of OSA (AHI ≥ 5) and no pre-existing CVD at baseline or within the first 2 years to account for unreported CVD at baseline. Exclusion criteria were central sleep apnea (central apneas ≥ 50% of total) or missing CVD outcome data. CVD was defined as diagnosis of stroke, heart failure, angina, myocardial infarction, coronary artery bypass grafting, or coronary angioplasty. Supplementary Figure S1 (online supplement) shows sample selection.

### Oximetry patterns

Oximetry signal processing is fully described in the online supplement. We calculated a total of 31 oximetry patterns across four analysis types: (1) *desaturation characteristics*, (2) *time series analysis*, (3) *power spectral density*, and (4) *non-linear analysis*. The oximetry patterns within each analysis approach are described below and represented graphical in Figure 1.

#### Desaturation characteristics

Oxygen Desaturation Index (ODI) is a commonly reported hypoxia measure of desaturation frequency which estimates the rate of oxygen desaturation events at fixed thresholds. A greater frequency and depth of desaturations will increase ODI values. We assessed ODI at thresholds of 2% (ODI2), 3% (ODI3), 4% (ODI4) and 5% (ODI5).

*Oxygen desaturation area* measures capture depth and duration of the desaturation curve. We have replicated a desaturation curve area measure which calculates the area of the curve following respiratory events [13]. The measure is given the name “hypoxic burden” and is calculated based on a patient-specific sampling window derived from the average desaturation response to respiratory events. The MATLAB code utilized to calculate this metric is available (https://github.com/pdechazal/Hypoxic-Burden). The final metric is expressed as the sum of individual desaturation areas divided by total sleep time (%min/h). As frequency and depth of the desaturations increase its value increases. It has been shown to be predictive of CVD mortality in population samples [13]. [24]

#### Time series analysis

The frequency of occurrence of SpO_2_ values across sleep can be analyzed based on their distribution pattern [17, 25]. We calculated the *mean, standard deviation, skewness* (distribution asymmetry) and *kurtosis* (distribution sharpness) for each participant. In OSA a greater variance in SpO_2_ values would be expected. Increasing OSA severity often leads to a longer tail below the mean value of the SpO_2_ values and hence an increased negative skewness. In OSA, a less sharp distribution would be expected leading to a lower value for kurtosis.

From the *cumulative frequency* of SpO_2_ values, the *median*, the *nadir* (minimum desaturation point) and the *percentage of time spent with SpO*_*2*_* ≤ X%* (TX) was calculated. For X we used eight SpO_2_ thresholds (80%, 82%, 84%, 86%, 88%, 90%, 92%, and 94%) to calculate T80, T82, T84, T86, T88, T92, T90, and T94. The TX values will increase as the frequency and depth of desaturation increases as is normally seen with increased OSA severity.

#### Power spectral density

The power spectral density (PSD) provides a frequency representation of the oximetry signal. An individual without OSA will typically have a steady oximetry value, resulting in a PSD with power concentrated near 0 Hz. An OSA patient with regular desaturations will have frequency components at the same frequency as the desaturations. An increased number and depth of desaturations will lead to more power in the associated frequency components. The full spectrum or the spectrum association with the possible range of frequency of desaturations (OSA spectrum) can be analyzed. We analyzed the full spectrum frequencies (0–0.1 Hz) [26] and OSA spectrum frequencies (0.0042–0.005 Hz). The OSA spectrum covered periodic desaturations in the range 15–180 events per minute (additional detail in online supplement). We calculated *mean, standard deviation, skewness,* and *kurtosis* of the full and OSA frequency range of the SpO_2_ signal PSD [17]. Higher values of mean, variance, and skewness and a lower kurtosis value are expected in OSA patients in both frequency ranges. We also calculated the spectral entropy of the full and OSA frequency range [27]. Spectral entropy quantifies the spectrum “flatness” with OSA patients expected to have higher values.

#### Non-linear analysis

The oximetry patterns described above use linear analysis methods. Non-linear analysis is an alternative approach producing parameters that have a non-linear relationship to the oximetry signal. We calculated *sample entropy* [28] and *central tendency measure* [29]. Sample entropy quantifies information related to the temporal order of oximetry signal values and quantifies the regularity of how often similar patterns are observed in the oximetry signal (Figure 1). To obtain sample entropy we resampled the SpO_2_ signal to 0.2 Hz and used the settings of window length *m* = 1, and a filtering level *r* = 0.25 as per previous analyses [30]. Generally in OSA the oximetry patterns are less regular and hence greater values of sample entropy would be expected. Central tendency measure (CTM) was also calculated from the 0.2 Hz resampled SpO_2_ signal and we used a circular region with a radius of 1% as per previous analyses [29]. For OSA, the less regular oximetry patterns result in lower CTM values than controls.

### Incident CVD

Our outcome was incident CVD. This was defined as first episode of angina, stroke, myocardial infarction, percutaneous transcutaneous angioplasty, coronary stent placement, coronary artery bypass grafting, chronic heart failure, coronary heart disease, or death due to CVD 2 years or more after baseline polysomnogram [23].

### Statistical analysis

Statistical analysis was performed in MATLAB R2017b (The Mathworks, Inc. Natick, Massachusetts, 2017). All oximetry variables were standardized (*z* scores) to allow direct comparison of oximetry patterns. Oximetry patterns were compared between those with and without incident CVD using independent *t*-tests. Relationship between AHI and oximetry patterns were assessed using Spearman’s rank correlation (online supplement).

We assessed oximetry patterns (continuous) during the sleep period of each patient and compared the associations with incident CVD using a series of Cox proportional hazard regression models (sleep-stage specific oximetry patterns in non-REM and REM sleep were additionally assessed and presented in the online supplement). We additionally derived separate models in men and women. Models were adjusted for covariates: age (continuous), race (Caucasian/other), smoking status (never/ever), BMI (continuous), sex (for non-sex stratified models). As AHI was highly correlated with many oximetry patterns (online supplement, Tables S1–S4) we were not able to adjust for OSA severity by the AHI in the models due to collinearity. Models with AHI as the predictor are shown for comparison. We graphically present the resulting hazard ratios (HR, and associated 95% confidence intervals, [95% CI]) for each oximetry pattern to compare the strength of the relationship with incident CVD. The HR is interpreted as the relative change in hazard associated with a one standard deviation increase of the oximetry parameter. As this analysis is exploratory in nature and involves multiple comparisons, we used the Benjamini-Hochberg procedure to adjust to adjust the false discovery rate [31]. The overall false discovery rate at 0.05 for oximetry patterns within a given analysis type. The exact p values can be found in the online supplement (Tables S6–S9).

## Results

### Sample characteristics

*n* = 2878 Participants with OSA met the inclusion criteria for analysis (Figure S1, online supplement). Sample characteristics are shown in Table 1. The sample were predominantly Caucasian race (88.3%) and women (51.8%). Length of study follow-up was median (interquartile range) 11.5 (2.8) years. Incident CVD occurred in *n* = 495 (17.2%). The mean AHI in the sample was in the moderate OSA range (20.7 ± 15.5 events/h). Nearly half the sample had mild OSA (46.3%), 34.0% moderate OSA and 19.7% severe OSA.

**Table 1.** Sample characteristics of the Sleep Heart Health Study cohort included in the analysis (*n* = 2878)

	Total *n* = 2878 (100%)		Men *n* = 1385 (48.1%)		Women *n* = 1493 (51.8%)	
Variables	No incident CVD *n* = 2383 (82.7%)	Incident CVD *n* = 495 (17.2%)	No incident CVD *n* = 1107 (79.9%)	Incident CVD *n* = 278 (20.1%)	No incident CVD *n* = 1276 (85.4%)	Incident CVD *n* = 217 (14.5%)
Age (years)	61.9 (10.1)	70.8 (9.3)	61.1 (9.9)	68.7 (9.7)	62.6 (10.3)	73.5 (8.2)
BMI (kg/m^2^)	28.8 (5.3)	28.7 (4.9)	28.8 (4.4)	29.1 (4.2)	28.8 (5.9)	28.3 (5.6)
Race						
Caucasian	2103 (88.3)	439 (88.7)	982 (88.7)	246 (88.5)	1121 (87.9)	193 (88.9)
Non-Caucasian	280 (11.7)	56 (11.3)	125 (11.3)	32 (11.5)	155 (12.1)	24 (11.1)
Smoking status, (current or former)	1235 (51.8)	273(55.2)	699 (63.1)	182 (65.5)	536(42.0)	91(41.9)
Total AHI (events/h)	20.4 (15.4)	22.3 (15.5)	24.0 (16.6)	25.7 (16.1)	17.3 (13.6)	18.0 (13.5)
OSA severity						
Mild	1140 (47.8)	192 (38.8)	384 (34.7)	84 (30.2)	756 (59.2)	108 (49.8)
Moderate	786 (33.0)	192 (38.8)	437 (39.5)	111 (39.9)	349 (27.4)	81 (37.3)
Severe	457 (19.2)	111 (22.4)	286 (25.8)	83 (29.9)	171 (13.4)	28 (12.9)

Data are presented as mean (standard deviation) for continuous variables, or *N* (%) for categorical variables.

### Oximetry patterns

Descriptive statistics of the 31 oximetry patterns across four analysis types in those without and with CVD are shown in Table 2. Comparison of oximetry patterns within NREM and REM sleep stages are shown in the online supplement (Table S5).

**Table 2.** Oximetry patterns in OSA in the Sleep Heart Health Study cohort

	All			Men			Women		
	No incident CVD	Incident CVD	*P* value	No incident CVD	Incident CVD	*P* value	No incident CVD	Incident CVD	*P* value
Pattern by type									
Respiratory events									
AHI (events/h)	20.39 ± 15.44	22.32 ± 15.47	0.011	23.96 ± 16.61	25.68 ± 16.04	0.121	17.29 ± 13.60	18.02 ± 13.54	0.462
Desaturation									
ODI2 (events/h)	18.14 ± 15.34	19.74 ± 15.22	0.035	21.46 ± 16.45	23.02 ± 15.99	0.157	15.26 ± 13.66	15.53 ± 13.02	0.789
ODI3 (events/h)	16.08 ± 14.87	17.42 ± 14.57	0.067	19.01 ± 16.04	20.58 ± 15.53	0.162	13.47 ± 13.23	13.37 ± 12.09	0.918
ODI4 (events/hr)	10.16 ± 12.58	11.03 ± 12.16	0.160	12.33 ± 14.03	13.65 ± 13.51	0.158	8.28 ± 10.82	7.67 ± 9.13	0.435
ODI5 (events/h)	6.69 ± 10.50	7.13 ± 9.85	0.392	8.23 ± 12.04	9.17 ± 11.31	0.240	5.35 ± 8.73	4.51 ± 6.73	0.178
Hypoxic Burden (%min/h)	52.99 ± 49.54	57.45 ± 43.31	0.063	63.92 ± 57.04	68.66 ± 48.63	0.203	43.50 ± 39.59	43.08 ± 29.70	0.881
Time series analysis									
Frequency distribution									
Mean (%SpO_2_)	94.61 ± 1.86	94.29 ± 1.97	0.001	94.19 ± 1.89	94.08 ± 2.03	0.384	94.98 ± 1.76	94.57 ± 1.85	0.002
SD (%SpO_2_)	1.51 ± 0.75	1.57 ± 0.62	0.105	1.60 ± 0.79	1.68 ± 0.64	0.131	1.43 ± 0.70	1.43 ± 0.55	0.942
Skewness (%SpO_2_)	‐0.91 ± 0.96	‐0.84 ± 0.91	0.140	‐0.83 ± 0.92	‐0.82 ± 0.91	0.890	‐0.99 ± 0.99	‐0.88 ± 0.92	0.119
Kurtosis (%SpO_2_)	7.05 ± 7.71	6.53 ± 5.25	0.155	6.62 ± 6.39	6.29 ± 5.16	0.428	7.41 ± 8.68	6.83 ± 5.35	0.338
Cumulative frequency distribution									
Median (%SpO_2_)	94.74 ± 1.87	94.40 ± 2.01	<0.001	94.32 ± 1.88	94.19 ± 2.06	0.313	95.10 ± 1.78	94.68 ± 1.90	0.001
Nadir (%SpO_2_)	85.02 ± 5.93	84.85 ± 5.31	0.558	84.49 ± 5.85	84.31 ± 5.30	0.652	85.48 ± 5.97	85.54 ± 5.25	0.895
T80 (%TST)	0.16 ± 1.51	0.07 ± 0.39	0.216	0.23 ± 2.09	0.09 ± 0.45	0.292	0.10 ± 0.65	0.05 ± 0.31	0.274
T82 (%TST)	0.25 ± 1.94	0.15 ± 0.67	0.250	0.35 ± 2.66	0.20 ± 0.80	0.353	0.17 ± 0.95	0.09 ± 0.44	0.223
T84 (%TST)	0.42 ± 2.62	0.30 ± 1.09	0.310	0.56 ± 3.51	0.40 ± 1.32	0.451	0.3 ± 1.46	0.17 ± 0.67	0.201
T86 (%TST)	0.76 ± 4.09	0.66 ± 2.32	0.587	0.96 ± 4.83	0.87 ± 2.80	0.783	0.59 ± 3.31	0.38 ± 1.46	0.360
T88 (%TST)	1.44 ± 5.94	1.63 ± 5.08	0.496	1.83 ± 6.92	2.20 ± 6.00	0.417	1.10 ± 4.91	0.91 ± 3.44	0.586
T90 (%TST)	3.38 ± 9.46	4.98 ± 12.21	0.001	4.43 ± 11.25	6.35 ± 14.03	0.016	2.48 ± 7.45	3.23 ± 9.07	0.183
T92 (%TST)	9.97 ± 18.10	14.32 ± 22.78	<0.001	12.74 ± 20.35	16.68 ± 24.18	0.006	7.57 ± 15.49	11.30 ± 20.46	0.002
T94 (%TST)	29.59 ± 30.39	35.42 ± 33.04	<0.001	35.99 ± 31.29	38.37 ± 33.05	0.263	24.04 ± 28.44	31.65 ± 32.65	<0.001
Power spectral density									
Full frequency band									
Mean ([SpO_2_]^2^)	17.07 ± 29.70	16.69 ± 18.58	0.787	20.02 ± 34.34	19.79 ± 20.29	0.914	14.51 ± 24.70	12.72 ± 15.22	0.304
SD ([%SpO_2_]^2^)	29.26 ± 52.27	30.19 ± 36.53	0.706	34.34 ± 60.86	35.72 ± 39.48	0.719	24.86 ± 42.98	23.11 ± 30.95	0.566
Skewness ([%SpO_2_]^2^)	2.90 ± 1.15	2.98 ± 1.16	0.147	2.82 ± 1.12	2.94 ± 1.20	0.098	2.97 ± 1.17	3.03 ± 1.10	0.476
Kurtosis ([%SpO_2_]^2^)	14.12 ± 9.23	14.56 ± 9.47	0.334	13.41 ± 8.92	14.28 ± 9.90	0.159	14.73 ± 9.45	14.92 ± 8.87	0.779
Spectral entropy (no units)	3.20 ± 0.20	3.14 ± 0.20	<0.001	3.18 ± 0.18	3.12 ± 0.20	<0.001	3.21 ± 0.21	3.16 ± 0.20	0.001
OSA frequency band									
Mean ([%SpO_2_]^2^)	28.44 ± 53.69	27.81 ± 33.84	0.800	33.95 ± 62.15	33.79 ± 38.11	0.969	23.67 ± 44.53	20.14 ± 25.45	0.256
SD ([%SpO_2_]^2^)	26.4 ± 58.52	28.37 ± 44.17	0.478	32.27 ± 68.57	35.23 ± 49.65	0.499	21.30 ± 47.54	19.59 ± 33.98	0.611
Skewness ([%SpO_2_]^2^)	0.89 ± 0.56	1.04 ± 0.67	<0.001	0.89 ± 0.57	1.09 ± 0.72	<0.001	0.89 ± 0.54	0.98 ± 0.59	0.033
Kurtosis ([%SpO_2_]^2^)	3.12 ± 1.73	3.61 ± 2.42	<0.001	3.13 ± 1.81	3.82 ± 2.79	<0.001	3.12 ± 1.66	3.35 ± 1.80	0.062
Spectral entropy (no units)	3.01 ± 0.14	2.96 ± 0.17	<0.001	2.99 ± 0.14	2.94 ± 0.18	<0.001	3.02 ± 0.14	2.98 ± 0.16	<0.001
Non-linear									
Sample entropy (no units)	0.63 ± 0.22	0.65 ± 0.23	0.116	0.67 ± 0.22	0.70 ± 0.24	0.141	0.60 ± 0.20	0.59 ± 0.19	0.661
Central Tendency Measure (no units)	0.44 ± 0.13	0.43 ± 0.12	0.009	0.42 ± 0.12	0.41 ± 0.12	0.321	0.47 ± 0.12	0.45 ± 0.12	0.096

Baseline oximetry patterns are shown grouped by analysis type. Respiratory event frequency (Apnea Hypopnea Index, AHI) is shown for comparison. Data are presented as mean ± standard deviation. *p* Values are from comparison (independent *t*-test) of those without and with incident cardiovascular disease (CVD) for the whole sample and men and women individually.

AHI, Apnea Hypopnea Index; ODI, Oxygen Desaturation Index; OSA, Obstructive Sleep Apnoea; SD, standard deviation; %SpO_2_, percent oxygen saturation; TST, total sleep time; TX, time spent below X% oxygen saturation.

### Comparative associations of oximetry patterns with incident CVD

**Figure 2.**
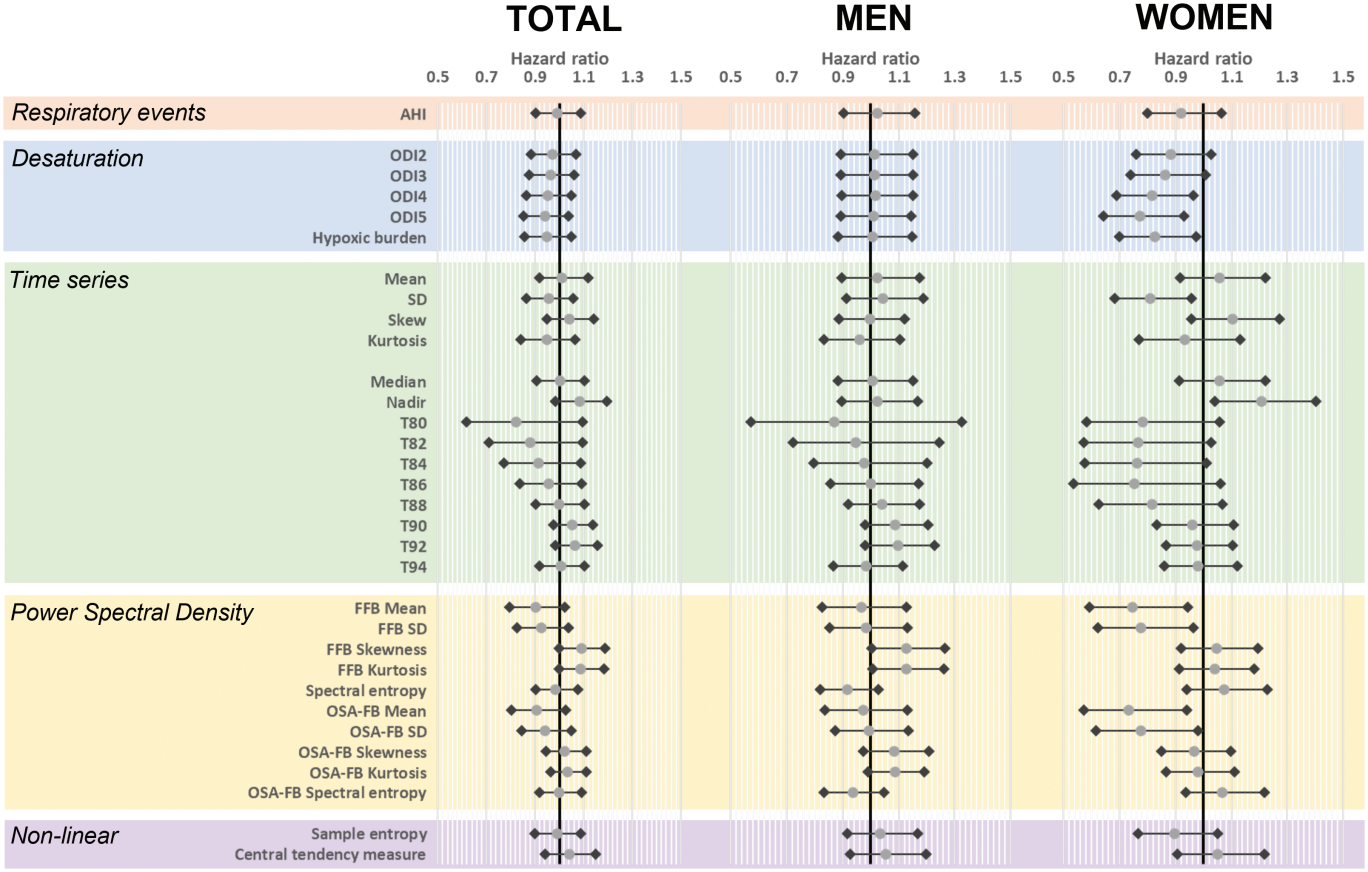
Comparison of oximetry patterns in adjusted Cox regression analyses for incident Cardiovascular Disease. Analysis is for the entire sample (*n* = 2878). Oximetry patterns models are presented for the whole sample and men and women separately. Respiratory events measured by the standard clinical measure of OSA severity, Apnea Hypopnea Index (AHI), is shown for comparison. Oximetry patterns are derived using four analysis approaches: desaturation characteristics, time series analysis, PSD, non-linear analysis. Models are adjusted for age, body mass index, race, sex, and smoking status. Grey circles represent hazard ratios with black diamonds the 95% confidence interval for the hazard ratio. FFB, full frequency band; ODI, oxygen desaturation index; OSA-FB, Obstructive Sleep Apnea frequency band.

We compared oximetry patterns for relationship to future CVD (Figure 2). AHI as a predictor variable is shown for comparison and was not significantly associated with incident CVD in this OSA sample. There were no significant associations between any of the oximetry patterns of the four analysis types and incident CVD in the total sample (Figure 2). Similar to the total sample, there were no significant associations between any of the oximetry patterns and incident CVD in the models in men (Figure 2).

Although none of the oximetry patterns reached statistical significance in women, some were suggestive (Figure 2). Within the desaturation characteristics ODI5 was significant for incident CVD (HR 0.77, 95% CI 0.64–0.83, *p* = 0.007) such that higher ODI5 was associated with lower hazard for CVD. For time series analysis, the standard deviation of the oximetry distribution was nominally significant (HR 0.81, 95% CI 0.68–0.97, *p* = 0.014), but not by the pre-specified significance level (*p* < 0.013). Similarly, for the cumulative distribution, the nadir (HR 1.2, 95% CI 1.04, 1.40, *p* = 0.01) was associated with CVD incidence. The mean and standard deviation of the full frequency band and mean of the OSA frequency band from power spectral analysis were also nominally significant. There was no association between non-linear oximetry patterns and incidence CVD in women.

A comparison of oximetry patterns specifically within NREM and REM sleep and for men and women are shown in the online supplement (Figures S2 and S3). The oximetry pattern relationships were similar to models for oximetry patterns across total sleep time.

## Discussion

We provide a direct comparison of OSA oximetry patterns in an OSA sample, derived using multiple analytic approaches, and their association with future CVD. Identifying oximetry patterns which relate to development of CVD in people with OSA but without baseline CVD could facilitate phenotyping for CVD risk in the sleep clinic and lead to precision approaches to OSA management. We found minimal evidence of a link to CVD for any individual or type of oximetry pattern. We found some evidence particular oximetry patterns having a stronger relationship to the occurrence of CVD in women with OSA.

A major strength of this analysis is the direct comparison of a range of oximetry patterns derived from different analytic approaches (Figure 1). The oximetry patterns considered included desaturation characteristics and times series analysis, which have been most widely investigated in relation to OSA, and additionally more novel analysis approaches of PSD and non-linear analysis to give a broad comparison. These latter types of analyses describe the frequency content of the oximetry signal and the regularity of the signal, respectively, and to our knowledge neither have been directly compared in relationship to CVD risk in OSA.

Within this OSA sample, we found no discriminatory ability of ODI, at any of the assessed thresholds. ODI 4%, rather than ODI 3%, has previously been shown to relate more strongly to cardiovascular outcomes in samples spanning no OSA to severe OSA [32]. In terms of desaturation characteristics, we also incorporated a desaturation curve area measure using the metric “hypoxic burden”, which has previously been shown to be a better predictor of cardiovascular mortality than AHI in whole population samples [13]. Within this OSA-only cohort, as with ODI, we did not find this measure useful for distinguishing those who would go on to develop CVD. However, in a recent analysis of a large clinical OSA cohort “hypoxic burden”, in addition to T90, was associated with a composite CV event and all-cause mortality outcome, although the hypoxic burden values appeared low for a more severe clinical cohort [33, 34]. Further investigation and comparison of desaturation type metrics in particular populations and against specific clinical outcomes may be warranted.

In terms of SpO_2_ distribution, it has been previously suggested that increased skewness towards low SpO_2_ and decreased kurtosis are features of OSA severity [17, 35, 36]. In relationship to future CVD in an OSA sample we did not find the shape of the distribution to have any discriminatory ability. Cumulative sleep time below 90% SpO_2_ (T90) is another commonly reported time series analysis variable shown to be predictive of mortality [10]. Examination of the cumulative histogram revealed no associations with incident CVD in an OSA sample. An increase in the nadir of oxygen saturation during total sleep was a protective factor in women. Although we looked at sleep time below a range of oxygen saturation levels, it should be noted that the sample had largely mild OSA and there was minimal data for many of these lower oxygen thresholds with average values of 0% time for many of these.

Power spectral analysis has been applied to as a method to identify OSA [26, 37]. In a cohort of nearly 1000 OSA patients with objective sleepiness data, it was recently shown that an increase in spectral power content of nocturnal SpO_2_ signals in the 0.015–0.035 Hz frequency band is associated with objective sleepiness on multiple sleep latency test [38]. To our knowledge, power spectral analysis of oximetry has not been previously assessed as a predictor of CVD risk. We found a suggestive association of increased mean and standard deviation of the full and OSA-band with decreased CVD risk within women only. Non-linear analysis patterns explicitly quantify information related to the temporal order of values in the oximetry signal. Measures quantifying how often similar patterns are observed in the oximetry signal applied to oximetry have been used for identifying OSA in adults and children and include entropy [28, 30, 39] and central tendency measure [29]. To our knowledge, entropy and central tendency measure has not previously been assessed for predicting CVD risk and we did not find evidence for its association with incident CVD in this OSA sample.

The lack of associations between OSA severity (AHI) and some oximetry patterns (traditional e.g. ODI, or more novel e.g. “hypoxic burden”) in the current analysis with incident CVD compared to previous studies using the SHHS data [10] is likely due to this analysis being specifically limited to the subset with OSA and no evidence of baseline CVD, and additionally differences in outcomes (incident CVD in the present study versus CVD or all-cause mortality in previous analyses) [10, 13]. In restricting to OSA-only we are truncating the range of these values to the higher end and hence a floor effect may explain the lack of association with future CVD in the current analysis. Other studies finding associations between metrics incorporating oxygen desaturation curve area and CVD outcomes or mortality have used a full spectrum of data from no disease to severe OSA [13, 14]. In terms of the potential advantage of more novel oxygen desaturation assessments compared to AHI another recent study has looked at this using the outcome of subclinical myocardial injury (cardiac troponin levels) [40]. This study found AHI, ODI, and novel metrics (incorporating oxygen desaturation area) were all similarly useful, with no advantage of the novel metrics, in discriminating those with elevated troponin [40]. A large OSA clinical cohort has found similar associations among ODI, T90, and “hypoxic burden” metrics in predicting major adverse CV events or all-cause mortality [34]. Our specific purpose was to compare the relative effect sizes of oximetry patterns, across multiple analysis approaches, within an OSA-only sample to determine if any patterns show a stronger signal for CVD risk for development as a prognostic marker within OSA clinical samples. A recent study in a sample with untreated OSA and comorbid CVD conducted a similar examination of clinical and novel oximetry patterns for association with CVD outcomes [41]. This study used oximetry data in the form of ODI, T90, mean, and nadir SpO_2_, and more novel desaturation duration and desaturation/re-saturation time ratio [41]. Similar to our findings, this study also found low prognostic value of oximetry data for a composite CVD outcome, although division of the outcome into different types of CVD did find some associations of oximetry patterns specifically with myocardial infarction and heart failure [41]. Together these studies suggest that within OSA samples, oximetry patterns do not consistently relate to composite CVD outcomes and future studies should explore associations with specific CVD outcomes based on likely mechanisms of disease.

Our study identified some suggestive oximetry signals in the models in women only. A stronger relationship between OSA and risk of CVD in women compared to men has been found in some cohorts [42, 43]. Sex differences in oxygen desaturation patterns following respiratory events in OSA have been noted [44] and therefore oximetry patterns related to CVD risk may differ between sexes. One data linkage study of nearly 10 years’ follow-up found the association between higher T90 and composite CVD outcome to be stronger for women [45].. The oximetry patterns associated with OSA in women in the current study however seem to be in the opposite and counterintuitive direction, in that oximetry patterns reflecting greater hypoxia associated with reduced risk of CVD. If this association is confirmed, one possible biological explanation is the concept of ischemic preconditioning [46]. The intermittent hypoxia in OSA leads to oxidative stress which at high levels promotes downstream pathologies, like CVD. But at more moderate or low levels this stimulus exposure promotes protective pathways for repair and survival [46]. Someone with this preconditioned response pathway in response to intermittent hypoxia through ongoing mild OSA may in some cases be in a better position to manage subsequent hypoxic insults. Nearly half of the sample in the present study had OSA in the mild severity range. The small signal detected for greater or more variable saturations being protective for CVD in these likely post-menopausal women, could be detecting benefits related to this ischemic preconditioning effect. Further investigation of the interplay between OSA severity, oximetry patterns, and CVD risk between the sexes is needed.

### Limitations

This analysis utilized available cohort data with baseline polysomnography and an average 15-year follow-up. Limitations to this data include lack of OSA treatment information which introduces error around the severity and duration of OSA experienced by patients. Although objective measurement of OSA by polysomnography was available at baseline, it is unclear for how long significant OSA had existed before that assessment which again influences the oximetry patterns and blurs any dose–response relationship with CVD. We have used a composite outcome for CVD for practical reasons, namely to increase numbers of participants with the outcome of interest. However, oximetry patterns may be uniquely linked to specific cardiovascular conditions [41]. There were under 500 cases of incident CVD in those with OSA in the sample. It is possible that we are underpowered to observe differences in oximetry patterns between those with OSA who do and do not go on to develop CVD as a composite outcome. We have utilized available data from a long-standing community-based cohort for this purpose as this is a unique opportunity to compare to 15-year follow-up CVD data. However we intended for our use of this available data to be hypothesis-generating and ultimately advocate for validation of these findings in independent samples with larger numbers of participants with different CVD considered as separate outcomes. In future studies it could be possible to examine much larger sample with polysomnography data from those who have attended clinical sleep laboratories and obtain follow-up data on CVD from data linkage and electronic medical records.

In conclusion, we comprehensively assessed multiple oximetry patterns derived using various analytic methodologies for relationships with future CVD in those with OSA. In an era where sleep medicine is looking to extract more from the multitude of sleep signals routinely collected for better prognostic tools, understanding aspects of the oximetry signal which could flag future CVD in the sleep clinic is highly desirable. In an initial exploration of baseline oximetry patterns in an OSA cohort we did not find strong links between any individual or type of oximetry pattern and incident CVD, and therefore no strong candidates for CVD risk phenotyping in the sleep clinic. Further work is needed to understand the complex relationship between OSA-related intermittent hypoxia patterns and development of CVD to further understanding of OSA phenotypes at risk of cardiovascular consequences.

## Supplementary Material

zsac179_suppl_Supplementary_Material
