# Supplementary material for: Comparative associations of oximetry patterns in Obstructive Sleep Apnea with incident cardiovascular disease
Source: Sleep. 2022 Jul 27;45(12):zsac179. doi: 10.1093/sleep/zsac179 (PMC9742894; doi:10.1093/sleep/zsac179)
Supplement: zsac179_suppl_Supplementary_Material [file zsac179_suppl_supplementary_material.docx]

**ONLINE SUPPLEMENT**

**Comparative associations of oximetry patterns in Obstructive Sleep Apnea with incident cardiovascular disease**

Kate Sutherland^1,2^, ­­­Nadi Sadr^1,3^, Yu Sun Bin^1,2^, Kristina Cook^1,2^, Hasthi U. Dissanayake^1,2^, Peter A. Cistulli^1,4^, and Philip de Chazal^1,2^

*^1^Sleep Research Group, Charles Perkins Centre. University of Sydney, Sydney, NSW, Australia; ^2^Northern Clinical School, Faculty of Medicine and Health, University of Sydney, Sydney, NSW, Australia; ^3^Department of Biomedical Informatics, Emory University, USA; ^4^Department of Respiratory & Sleep Medicine, Royal North Shore Hospital, St Leonards, NSW, Australia.^5^School of Biomedical Engineering, University of Sydney, Sydney, NSW, Australia.*

Corresponding author:

Prof Philip de Chazal

Sleep Research Group, Level 3

Charles Perkins Centre

University of Sydney

Email: philip.dechazal@sydney.edu.au

Phone: +61 2 911 41528

**METHODS**

**Sleep Heart Health Study**

The Sleep Heart Health Study (SHHS) was a multi-centered cohort study investigating the cardiovascular consequences of sleep-disordered breathing. The SHHS recruited from a number of ‘parent cohorts’ established for the purpose of investigating cardiovascular risk factors. Baseline data collection was between 1995 and 1998 and participants were followed until May 2011[1, 2]. Briefly, participants aged over 40 years were recruited if they had no previous sleep apnoea treatment, tracheostomy, or current home oxygen therapy. The SHHS data includes questionnaire data, polysomnography data, detailed respiratory and sleep staging events, and cardiovascular outcome data.

Cardiovascular outcomes were collected for the SHHS through a combination of direct participant contact at intervals of 1 to 4 years, surveying death certificates and discharge information from local hospitals, and mailings to study participants. All potential outcomes were further investigated and adjudicated using defined protocols, which included physician review of abstracted data [3].

The selection process for inclusion of SHHS participants in the current analysis are shown **in Figure S1**.

**Polysomnographic scoring**

We used the available event scoring and sleep staging files for the SHHS. We used the respiratory events scored using the AASM 2007 scoring rules (hypopnea scored when the signal maximum drop by ≥30% for ≥10 seconds in association with either ≥3% arterial oxygen desaturation or an arousal) [4] and selected all events that fulfilled these criteria. The sleep staging was performed using the R&K rules on 30 second epochs [5]. The SHHS supplemental tables provided AHI for the full recording which we used. AHI for the NREM and REM sleep states were calculated from the event scorings and sleep staging files.

### **Oximetry signal processing**

Baseline overnight polysomnography included finger-tip pulse oximetry signals (SpO_2_, peripheral capillary oxygen saturation) with a sampling rate of 1Hz. Artefact samples of the oximetry were detected by identifying samples outside the physiologically plausible range of 50-100% and excluded from analysis. Oximetry patterns were calculated across total sleep time, and within non-REM and REM sleep time.

**Power spectral density analysis**

Our SpO2 signal is sampled at 1Hz so 10-minute windows provides a frequency resolution of 1/600s= 0.00167Hz. The SpO2 signal PSD analysis provides information on the frequency content of the periodic desaturations. For OSA patients we have decided for our purposes we are interested in detecting periodic desaturations in the range 15 to 180 events per minute which corresponds to a frequency range of 0.0042-0.05Hz. The windows of data were overlapped by 30 seconds to align with the hypnogram scorings. The PSD was calculated with the Lomb periodogram to allow for the missing values in the oximetry signal.


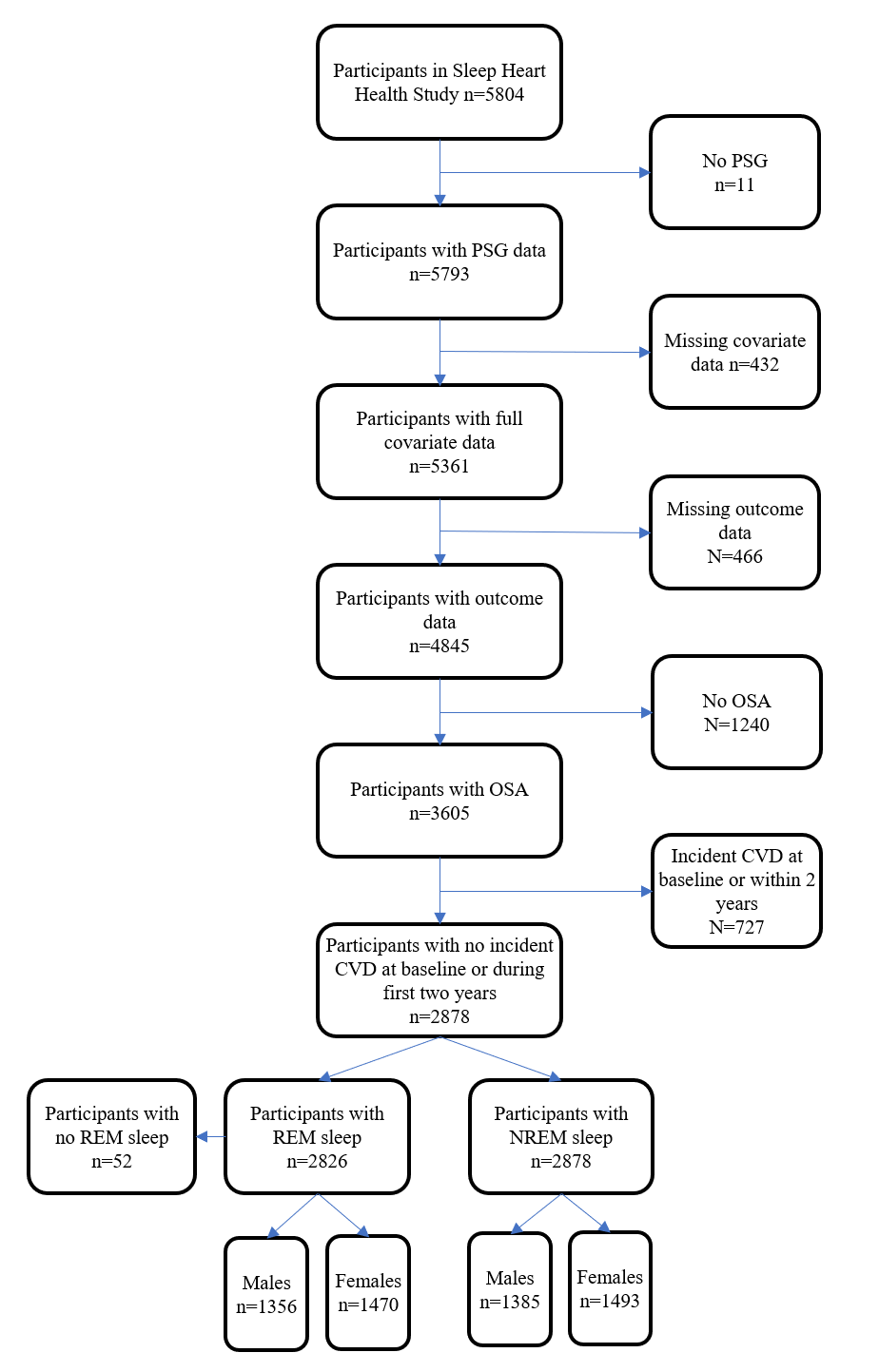


**Figure S1. Flow chart for the study sample identified for inclusion from the Sleep Heart Health Study cohort database**. Inclusion criteria for this analysis was the presence of Obstructive Sleep Apnoea (Apnoea-Hypopnea Index [AHI] ≥5 events/hour) who did not have evidence of cardiovascular disease at baseline (or in the first two years of follow-up). Other requirements were available covariate data and polysomnographic data. N=2877 were included in the current analysis (n=1385 men, n=1493 women).

**RESULTS**

*Relationships between AHI and oximetry pattern variables*

Correlations (Spearman’s rank) are shown between Apnoea-Hypopnea Index (AHI) and oximetry patterns by each of the four oximetry analysis methods (desaturation characteristics, time series analysis, power spectral density, and non-linear analysis) in **Tables S1-S4**.

*Comparative associations of oximetry patterns in NREM and REM sleep with incident CVD*

Associations of all of the oximetry pattern variables by NREM and REM sleep with incident CVD are shown in **Figure S2**. NREM and REM sleep oximetry patterns are also given for men and women separately in **Figure S3**.

**Table S1: Relationships between Apnoea-Hypopnea Index (AHI) and oxygen patterns (desaturation characteristics analysis).** Correlation coefficients (Spearman’s rank) are shown for the relationships between AHI and the oximetry desaturation characteristics measures investigated in this analysis.

|  | **AHI** | **ODI2** | **ODI3** | **ODI4** | **ODI5** | **Hypoxic burden** |
| --- | --- | --- | --- | --- | --- | --- |
| **AHI** | 1.00 | 0.97 | 0.94 | 0.89 | 0.84 | 0.92 |
| **ODI2** | 0.97 | 1.00 | 0.99 | 0.95 | 0.89 | 0.93 |
| **ODI3** | 0.94 | 0.99 | 1.00 | 0.97 | 0.92 | 0.93 |
| **ODI4** | 0.89 | 0.95 | 0.97 | 1.00 | 0.98 | 0.90 |
| **ODI5** | 0.84 | 0.89 | 0.92 | 0.98 | 1.00 | 0.87 |
| **Hypoxic burden** | 0.92 | 0.93 | 0.93 | 0.90 | 0.87 | 1.00 |

**Table S2: Relationships between Apnoea-Hypopnea Index (AHI) and oxygen patterns (time series analysis).** Correlation coefficients (Spearman’s rank) are shown for the relationships between AHI and the oximetry time series analysis measures investigated in this analysis.

|  |  |  | *Frequency Distribution* | | | | *Cumulative frequency* | | | | | | | | | |
| --- | --- | --- | --- | --- | --- | --- | --- | --- | --- | --- | --- | --- | --- | --- | --- | --- |
|  |  | **AHI** | **Mean** | **SD** | **Skewness** | **Kurtosis** | **Median** | **Nadir** | **T80** | **T82** | **T84** | **T86** | **T88** | **T90** | **T92** | **T94** |
|  | **AHI** | 1.00 | 0.36 | 0.64 | -0.26 | 0.21 | -0.35 | -0.58 | 0.38 | 0.44 | 0.49 | 0.54 | 0.58 | 0.60 | 0.57 | 0.44 |
| *Frequency Distribution* | **Mean** | -0.39 | 1.00 | -0.57 | -0.18 | 0.13 | 0.98 | 0.54 | -0.33 | -0.38 | -0.42 | -0.49 | -0.60 | -0.73 | -0.87 | -0.97 |
|  | **SD** | 0.68 | -0.57 | 1.00 | -0.29 | 0.14 | -0.51 | -0.80 | 0.55 | 0.63 | 0.70 | 0.76 | 0.81 | 0.85 | 0.81 | 0.65 |
|  | **Skewness** | -0.23 | -0.18 | -0.29 | 1.00 | -0.84 | -0.25 | 0.52 | -0.40 | -0.47 | -0.49 | -0.51 | -0.47 | -0.34 | -0.13 | 0.12 |
|  | **Kurtosis** | 0.16 | 0.13 | 0.14 | -0.84 | 1.00 | 0.17 | -0.53 | 0.34 | 0.41 | 0.44 | 0.47 | 0.43 | 0.30 | 0.09 | -0.11 |
| *Cumulative frequency* | **Median** | -0.35 | 0.98 | -0.51 | -0.25 | 0.17 | 1.00 | 0.48 | -0.28 | -0.32 | -0.37 | -0.43 | -0.54 | -0.67 | -0.83 | -0.94 |
|  | **Nadir** | -0.58 | 0.54 | -0.80 | 0.52 | -0.53 | 0.48 | 1.00 | -0.62 | -0.73 | -0.82 | -0.90 | -0.93 | -0.88 | -0.76 | -0.58 |
|  | **T80** | 0.38 | -0.33 | 0.55 | -0.40 | 0.34 | -0.28 | -0.62 | 1.00 | 0.84 | 0.73 | 0.64 | 0.57 | 0.51 | 0.43 | 0.31 |
|  | **T82** | 0.44 | -0.38 | 0.63 | -0.47 | 0.41 | -0.32 | -0.73 | 0.84 | 1.00 | 0.87 | 0.76 | 0.68 | 0.60 | 0.50 | 0.37 |
|  | **T84** | 0.49 | -0.42 | 0.70 | -0.49 | 0.44 | -0.37 | -0.82 | 0.73 | 0.87 | 1.00 | 0.88 | 0.78 | 0.69 | 0.58 | 0.43 |
|  | **T86** | 0.54 | -0.49 | 0.76 | -0.51 | 0.47 | -0.43 | -0.90 | 0.64 | 0.76 | 0.88 | 1.00 | 0.91 | 0.81 | 0.67 | 0.51 |
|  | **T88** | 0.58 | -0.60 | 0.81 | -0.47 | 0.43 | -0.54 | -0.93 | 0.57 | 0.68 | 0.78 | 0.91 | 1.00 | 0.92 | 0.79 | 0.62 |
|  | **T90** | 0.60 | -0.73 | 0.85 | -0.34 | 0.30 | -0.67 | -0.88 | 0.51 | 0.60 | 0.69 | 0.81 | 0.92 | 1.00 | 0.92 | 0.77 |
|  | **T92** | 0.57 | -0.87 | 0.81 | -0.13 | 0.09 | -0.83 | -0.76 | 0.43 | 0.50 | 0.58 | 0.67 | 0.79 | 0.92 | 1.00 | 0.92 |
|  | **T94** | 0.44 | -0.97 | 0.65 | 0.12 | -0.11 | -0.94 | -0.58 | 0.31 | 0.37 | 0.43 | 0.51 | 0.62 | 0.77 | 0.92 | 1.00 |

**Table S3: Relationships between Apnoea-Hypopnea Index (AHI) and oxygen patterns (power spectral density analysis).** Correlation coefficients (Spearman’s rank) are shown for the relationships between AHI and the oximetry power spectral density analysis measures investigated in this analysis.

|  |  |  | *Full frequency band* | | | | | *OSA frequency band* | | | | |
| --- | --- | --- | --- | --- | --- | --- | --- | --- | --- | --- | --- | --- |
|  |  | **AHI** | **Mean** | **SD** | **Skewness** | **Kurtosis** | **Spectral entropy** | **Mean** | **SD** | **Skewness** | **Kurtosis** | **Spectral entropy** |
|  | **AHI** | 1.00 | 0.80 | 0.72 | -0.42 | -0.43 | -0.05 | 0.82 | 0.75 | -0.02 | 0.02 | -0.18 |
| *Full frequency band* | **Mean** | 0.80 | 1.00 | 0.97 | -0.28 | -0.32 | -0.36 | 0.99 | 0.96 | 0.18 | 0.16 | -0.41 |
|  | **SD** | 0.72 | 0.97 | 1.00 | -0.11 | -0.15 | -0.53 | 0.94 | 0.95 | 0.29 | 0.25 | -0.50 |
|  | **Skewness** | -0.42 | -0.28 | -0.11 | 1.00 | 0.99 | -0.41 | -0.39 | -0.33 | 0.28 | 0.30 | 0.03 |
|  | **Kurtosis** | -0.43 | -0.32 | -0.15 | 0.99 | 1.00 | -0.33 | -0.42 | -0.38 | 0.20 | 0.23 | 0.12 |
|  | **Spectral entropy** | -0.05 | -0.36 | -0.53 | -0.41 | -0.33 | 1.00 | -0.29 | -0.45 | -0.58 | -0.42 | 0.81 |
| *OSA frequency band* | **Mean** | 0.82 | 0.99 | 0.94 | -0.39 | -0.42 | -0.29 | 1.00 | 0.97 | 0.14 | 0.12 | -0.40 |
|  | **SD** | 0.75 | 0.96 | 0.95 | -0.33 | -0.38 | -0.45 | 0.97 | 1.00 | 0.30 | 0.23 | -0.60 |
|  | **Skewness** | -0.02 | 0.18 | 0.29 | 0.28 | 0.20 | -0.58 | 0.14 | 0.30 | 1.00 | 0.92 | -0.62 |
|  | **Kurtosis** | 0.02 | 0.16 | 0.25 | 0.30 | 0.23 | -0.42 | 0.12 | 0.23 | 0.92 | 1.00 | -0.40 |
|  | **Spectral entropy** | -0.18 | -0.41 | -0.50 | 0.03 | 0.12 | 0.81 | -0.40 | -0.60 | -0.62 | -0.40 | 1.00 |

**Table S4: Relationships between Apnoea-Hypopnea Index (AHI) and oxygen patterns (non-linear analysis).** Correlation coefficients (Spearman’s rank) are shown for the relationships between AHI and the oximetry non-linear analysis measures investigated in this analysis.

|  | **AHI** | **Sample entropy** | **Central tendency measure** |
| --- | --- | --- | --- |
| **AHI** | 1.00 | 0.73 | -0.64 |
| **Sample entropy** | 0.73 | 1.00 | -0.68 |
| **Central tendency measure** | -0.64 | -0.68 | 1.00 |

**Table S5. Oximetry parameters in OSA in the Sleep Heart Health Study cohort.** Oximetry parameters are shown grouped by type, and presented for NREM and REM sleep. Data are presented as Mean ± Standard Deviation.

|  | **All** | | **Men** | | **Women** | |
| --- | --- | --- | --- | --- | --- | --- |
| **Parameters by type** | **NREM** | **REM** | **NREM** | **REM** | **NREM** | **REM** |
| *Respiratory Events* |  |  |  |  |  |  |
| **AHI** (events/hr) | 18.2 ± 16.3 | 31.0 ± 19.8 | 22.5 ± 17.5 | 31.8 ± 19.8 | 14.3 ± 14.0 | 30.4 ± 19.8 |
| *Desaturation* |  |  |  |  |  |  |
| **ODI2** (events/hr) | 15.9 ± 16.1 | 28.6 ± 19.9 | 19.9 ± 17.3 | 29.4 ± 19.8 | 12.2 ± 13.8 | 27.8 ± 20.0 |
| **ODI3** (events/hr) | 13.8 ± 15.4 | 26.4 ± 19.7 | 17.3 ± 16.7 | 27.3 ± 19.6 | 10.4 ± 13.2 | 25.7 ± 19.8 |
| **ODI4** (events/hr) | 8.5 ± 12.7 | 17.7 ± 17.9 | 11.2 ± 14.4 | 18.6 ± 18.0 | 6.1 ± 10.3 | 16.8 ± 17.8 |
| **ODI5** (events/hr) | 5.4 ± 10.4 | 12.3 ± 15.7 | 7.3 ± 12.1 | 13.2 ± 15.9 | 3.7 ± 8.0 | 11.6 ± 15.4 |
| **Hypoxic Burden** (%min/hr) | 53.8 ± 48.7 | 52.9 ± 48.9 | 64.9 ± 55.5 | 63.7 ± 56.1 | 43.4 ± 38.3 | 42.8 ± 38.6 |
| *Time series analysis* |  |  |  |  |  |  |
| Frequency distribution |  |  |  |  |  |  |
| **Mean** (%SpO_2_) | 94.6 ± 1.8 | 94.3 ± 2.4 | 94.2 ± 1.9 | 93.9 ± 2.5 | 95.0 ± 1.7 | 94.7 ± 2.2 |
| **SD** (%SpO_2_) | 1.3 ± 0.6 | 1.9 ± 1.2 | 1.4 ± 0.6 | 2.0 ± 1.2 | 1.2 ± 0.5 | 1.9 ± 1.1 |
| **Skewness** (%SpO_2_) | -0.6 ± 0.9 | -0.8 ± 0.8 | -0.6 ± 0.9 | -0.7 ± 0.7 | -0.6 ± 0.9 | -0.9 ± 0.8 |
| **Kurtosis** (%SpO_2_) | 6.2 ± 8.0 | 4.9 ± 3.8 | 6.0 ± 6.6 | 4.5 ± 3.2 | 6.5 ± 9.0 | 5.2 ± 4.3 |
| Cumulative frequency distribution |  |  |  |  |  |  |
| **Median** (%SpO_2_) | 94.7 ± 1.9 | 94.6 ± 2.4 | 94.3 ± 1.9 | 94.1 ± 2.5 | 95.0 ± 1.8 | 95.0 ± 2.2 |
| **Nadir** (%SpO_2_) | 86.8 ± 5.0 | 86.2 ± 5.9 | 86.0 ± 5.0 | 85.8 ± 5.8 | 87.5 ± 4.9 | 86.6 ± 6.0 |
| **T80** (%TST) | 0.1 ± 1.2 | 0.6 ± 3.6 | 0.1 ± 1.6 | 0.7 ± 4.5 | 0.0 ± 0.4 | 0.4 ± 2.4 |
| **T82** (%TST) | 0.1 ± 1.5 | 0.9 ± 4.6 | 0.2 ± 2.1 | 1.1 ± 5.7 | 0.1 ± 0.6 | 0.6 ± 3.3 |
| **T84** (%TST) | 0.2 ± 2.0 | 1.4 ± 6.0 | 0.3 ± 2.8 | 1.7 ± 7.3 | 0.1 ± 0.9 | 1.1 ± 4.5 |
| **T86** (%TST) | 0.4 ± 3.5 | 2.2 ± 7.9 | 0.6 ± 4.0 | 2.7 ± 9.2 | 0.3 ± 2.9 | 1.8 ± 6.4 |
| **T88** (%TST) | 1.0 ± 5.3 | 3.9 ± 10.6 | 1.3 ± 6.2 | 4.6 ± 12.0 | 0.6 ± 4.3 | 3.2 ± 9.1 |
| **T90** (%TST) | 2.8 ± 9.5 | 7.5 ± 15.1 | 3.9 ± 11.4 | 8.9 ± 16.9 | 1.8 ± 7.2 | 6.1 ± 13.2 |
| **T92** (%TST) | 9.7 ± 19.0 | 15.4 ± 22.4 | 12.5 ± 21.2 | 18.4 ± 24.3 | 7.1 ± 16.3 | 12.7 ± 20.0 |
| **T94** (%TST) | 30.2 ± 31.8 | 32.9 ± 30.4 | 36.1 ± 32.5 | 38.6 ± 31.0 | 24.6 ± 30.0 | 27.7 ± 28.9 |
| *Power spectral density* |  |  |  |  |  |  |
| Full frequency band |  |  |  |  |  |  |
| **Mean** ([SpO_2_]^2^) | 11.4 ± 21.2 | 36.3 ± 69.6 | 14.4 ± 26.1 | 39.4 ± 72.9 | 8.6 ± 14.8 | 33.5 ± 66.3 |
| **SD** ([%SpO_2_]^2^) | 19.3 ± 40.8 | 69.3 ± 140.8 | 24.8 ± 48.7 | 76.6 ± 155.4 | 14.3 ± 30.9 | 62.4 ± 125.5 |
| **Skewness** ([%SpO_2_]^2^) | 3.0 ± 1.2 | 2.6 ± 1.0 | 2.9 ± 1.2 | 2.6 ± 1.0 | 3.0 ± 1.3 | 2.6 ± 1.0 |
| **Kurtosis** ([%SpO_2_]^2^) | 14.9 ± 9.9 | 11.1 ± 7.8 | 14.0 ± 9.7 | 11.0 ± 7.3 | 15.7 ± 10.3 | 11.3 ± 7.8 |
| **Spectral entropy** (no units) | 3.3 ± 0.2 | 3.0 ± 0.5 | 3.3 ± 0.2 | 3.9 ± 0.5 | 3.3 ± 0.2 | 3.0 ± 0.4 |
| OSA frequency band |  |  |  |  |  |  |
| **Mean** ([%SpO_2_]^2^) | 19.5 ± 39.7 | 61.8 ± 125.6 | 25.2 ± 48.4 | 67.9 ± 134.1 | 14.3 ± 28.4 | 56.2 ± 116.9 |
| **SD** ([%SpO_2_]^2^) | 18.6 ± 51.2 | 72.1 ± 168.5 | 25.0 ± 60.2 | 82.2 ± 192.9 | 12.6 ± 40.2 | 62.7 ± 141.7 |
| **Skewness** ([%SpO_2_]^2^) | 0.9 ± 0.7 | 1.3 ± 0.6 | 1.0 ± 0.8 | 1.4 ± 0.6 | 0.8 ± 0.6 | 1.3 ± 0.6 |
| **Kurtosis** ([%SpO_2_]^2^) | 3.5 ± 2.3 | 4.3 ± 2.5 | 3.7 ± 2.6 | 4.3 ± 2.6 | 3.3 ± 2.0 | 4.2 ± 2.5 |
| **Spectral entropy** (no units) | 3.1 ± 0.2 | 2.8 ± 0.4 | 3.0 ± 0.2 | 2.9 ± 0.4 | 3.1 ± 0.1 | 2.8 ± 0.4 |
| *Non-linear* |  |  |  |  |  |  |
| **Sample entropy** (no units) | 0.6 ± 0.2 | 0.8 ± 0.3 | 0.7 ± 0.2 | 0.8 ± 0.3 | 0.6 ± 0.2 | 0.7 ± 0.3 |
| **Central tendency measure** (no units) | 0.36 ± 0.10 | 0.08 ±0.04 | 0.34 ± 0.10 | 0.07 ± 0.04 | 0.39 ± 0.10 | 0.08 ± 0.04 |

AHI – Apnoea Hypopnoea Index; ODI – Oxygen Desaturation Index; OSA – Obstructive Sleep Apnoea; SD – Standard Deviation; %SpO_2_ – percent oxygen saturation; TST – Total Sleep Time; TX – Time spent below X% oxygen saturation

**Table S6. Comparison of oximetry patterns in adjusted Cox regression analyses for incident cardiovascular disease**. Analysis is for the entire sample (N=2878). Oximetry patterns models are presented for the whole sample and men and women separately. Respiratory events measured by the standard clinical measure of OSA severity, Apnoea Hypopnea Index (AHI), is shown for comparison. Oximetry patterns are derived using four analysis approaches: desaturation characteristics, time series analysis, power spectral density, non-linear analysis. Models are adjusted for age, body mass index, race, sex, and smoking status. Data presented is hazard ratio (95% confidence interval) and p value. This data is presented graphically in the main paper in **Figure 2**.

*FFB – full frequency band; ODI – oxygen desaturation index; OSA-FB – Obstructive Sleep Apnoea Frequency Band*

|  | **All** | | **Men** | | **Women** | |
| --- | --- | --- | --- | --- | --- | --- |
| **Parameters by type** | **Hazard ratio (95% CI)** | **P value** | **Hazard ratio (95% CI)** | **P value** | **Hazard ratio (95% CI)** | **P value** |
| *Respiratory Events* |  |  |  |  |  |  |
| **AHI** (events/hr) | 0.99  (0.90, 1.09) | 0.865 | 1.02  (0.90, 1.16) | 0.707 | 0.92  (0.80, 1.06) | 0.262 |
| *Desaturation* |  |  |  |  |  |  |
| **ODI2** (events/hr) | 0.97  (0.88, 1.07) | 0.553 | 1.01  (0.89, 1.15) | 0.821 | 0.88  (0.76, 1.03) | 0.108 |
| **ODI3** (events/hr) | 1.06  (0.88, 1.06) | 0.452 | 1.01  (0.89, 1.15) | 0.831 | 0.86  (0.74, 1.01) | 0.065 |
| **ODI4** (events/hr) | 0.95  (0.87, 1.05) | 0.316 | 1.02  (0.90, 1.15) | 0.783 | 0.82  (0.69, 0.96) | 0.017 |
| **ODI5** (events/hr) | 0.94  (0.85, 1.04) | 0.217 | 1.01  (0.89, 1.15) | 0.844 | 0.78  (0.64, 0.93) | 0.007 |
| **Hypoxic Burden** (%min/hr) | 0.95  (0.86, 1.05) | 0.311 | 1.01  (0.88, 1.15) | 0.913 | 0.83  (0.70, 0.97) | 0.023 |
| *Time series analysis* |  |  |  |  |  |  |
| Frequency distribution |  |  |  |  |  |  |
| **Mean** (%SpO_2_) | 1.01  (0.92, 1.12) | 0.797 | 1.03  (0.90, 1.17) | 0.710 | 1.06  (0.92, 1.22) | 0.435 |
| **SD** (%SpO_2_) | 0.96  (0.86, 1.06) | 0.382 | 1.04  (0.91, 1.19) | 0.530 | 0.81  (0.68, 0.96) | 0.014 |
| **Skewness** (%SpO_2_) | 1.04  (0.95, 1.14) | 0.373 | 1.0  (0.89, 1.12) | 0.971 | 1.10  (0.96, 1.27) | 0.172 |
| **Kurtosis** (%SpO_2_) | 0.95  (0.84, 1.07) | 0.376 | 0.96  (0.83, 1.11) | 0.578 | 0.93  (0.77, 1.13) | 0.479 |
| Cumulative frequency distribution |  |  |  |  |  |  |
| **Median** (%SpO_2_) | 1.00  (0.91, 1.11) | 0.969 | 1.01  (0.88, 1.15) | 0.890 | 1.06  (0.92, 1.22) | 0.449 |
| **Nadir** (%SpO_2_) | 1.08  (0.98, 1.20) | 0.103 | 1.03  (0.90, 1.17) | 0.713 | 1.21  (1.04, 1.40) | 0.013 |
| **T80** (%TST) | 0.82  (0.62, 1.09) | 0.182 | 0.87  (0.57, 1.32) | 0.518 | 0.78  (0.58, 1.06) | 0.112 |
| **T82** (%TST) | 0.88  (0.71, 1.20) | 0.255 | 0.948  (0.72, 1.24) | 0.702 | 0.77  (0.57, 1.03) | 0.074 |
| **T84** (%TST) | 0.92  (0.77, 1.09) | 0.315 | 0.98  (0.80, 1.20) | 0.834 | 0.76  (0.58, 1.01) | 0.061 |
| **T86** (%TST) | 0.96  (0.84, 1.09) | 0.510 | 1.00  (0.86, 1.17) | 0.972 | 0.75  (0.54, 1.06) | 0.105 |
| **T88** (%TST) | 1.00  (0.90, 1.10) | 0.968 | 1.04  (0.92, 1.18) | 0.513 | 0.82  (0.63, 1.07) | 0.138 |
| **T90** (%TST) | 1.05  (0.98, 1.14) | 0.176 | 1.09  (0.98, 1.20) | 0.109 | 0.96  (0.83, 1.11) | 0.587 |
| **T92** (%TST) | 1.07  (0.98, 1.16) | 0.122 | 1.10  (0.98, 1.23) | 0.101 | 0.98  (0.87, 1.10) | 0.723 |
| **T94** (%TST) | 1.01  (0.92, 1.10) | 0.890 | 0.98  (0.87, 1.12) | 0.796 | 0.98  (0.86, 1.12) | 0.784 |
| *Power spectral density* |  |  |  |  |  |  |
| Full frequency band |  |  |  |  |  |  |
| **Mean** ([SpO_2_]^2^) | 0.90  (0.80, 1.02) | 0.111 | 0.97  (0.83, 1.13) | 0.669 | 0.75  (0.59, 0.95) | 0.015 |
| **SD** ([%SpO_2_]^2^) | 0.93  (0.83, 1.04) | 0.189 | 0.98  (0.85, 1.13) | 0.817 | 0.78  (0.62, 0.96) | 0.022 |
| **Skewness** ([%SpO_2_]^2^) | 1.09  (1.0, 1.19) | 0.051 | 1.12  (1.00, 1.27) | 0.043 | 1.05  (0.92, 1.20) | 0.466 |
| **Kurtosis** ([%SpO_2_]^2^) | 1.09  (1.0, 1.18) | 0.053 | 1.13  (1.01, 1.26) | 0.035 | 1.04  (0.91, 1.18) | 0.550 |
| **Spectral entropy** (no units) | 0.98  (0.90, 1.08) | 0.735 | 0.92  (0.82, 1.03) | 0.141 | 1.08  (0.94, 1.23) | 0.288 |
| OSA frequency band |  |  |  |  |  |  |
| **Mean** ([%SpO_2_]^2^) | 0.91  (0.80, 1.03) | 0.126 | 0.97  (0.83, 1.13) | 0.727 | 0.73  (0.57, 0.94) | 0.014 |
| **SD** ([%SpO_2_]^2^) | 0.94  (0.85, 1.05) | 0.282 | 1.0  (0.87, 1.13) | 0.952 | 0.78  (0.62, 0.98) | 0.034 |
| **Skewness** ([%SpO_2_]^2^) | 1.02  (0.94, 1.11) | 0.563 | 1.10  (0.98, 1.21) | 0.131 | 0.97  (0.85, 1.10) | 0.617 |
| **Kurtosis** ([%SpO_2_]^2^) | 1.04  (0.97, 1.11) | 0.324 | 1.09  (0.99, 1.19) | 0.07 | 0.98  (0.87, 1.11) | 0.762 |
| **Spectral entropy** (no units) | 1.00  (0.92, 1.09) | 0.982 | 0.94  (0.83, 1.05) | 0.257 | 1.07  (0.94, 1.22) | 0.319 |
| *Non-linear* |  |  |  |  |  |  |
| **Sample entropy** (no units) | 0.99  (0.89, 1.09) | 0.843 | 1.05  (0.92, 1.17) | 0.573 | 0.90  (0.77, 1.05) | 0.181 |
| **Central tendency measure** (no units) | 1.04  (0.94, 1.15) | 0.431 | 1.05  (0.93, 1.20) | 0.427 | 1.05  (0.91, 1.22) | 0.511 |

**
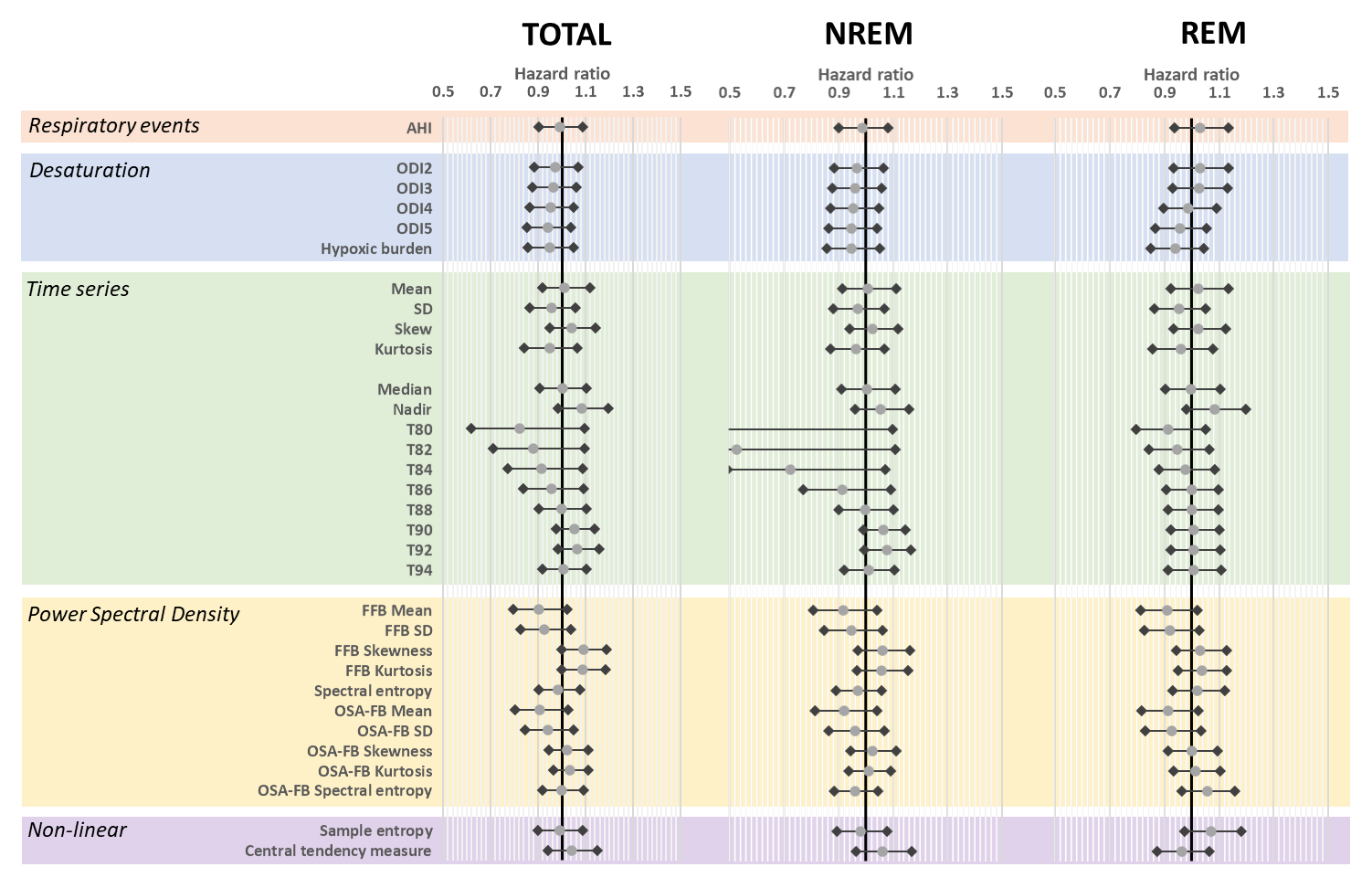
**

**Figure S2. Comparison of oximetry patterns in adjusted Cox regression analyses for incident Cardiovascular Disease**. Analysis is for the entire sample (N=2863) and oximetry patterns are measured separately within NREM and REM sleep (oximetry patterns for total sleep time are shown for comparison). Respiratory events measured by the standard clinical measure of OSA severity, Apnoea Hypopnea Index (AHI), is shown for comparison. Oximetry patterns are derived using four analysis approaches: desaturation characteristics, time series analysis, power spectral density, non-linear analysis. Models are adjusted for age, body mass index, race, sex, and smoking status. Grey circles represent hazard ratios with black diamonds the 95% confidence interval for the hazard ratio.

*FFB – full frequency band; ODI – oxygen desaturation index; OSA-FB – Obstructive Sleep Apnoea Frequency Band; SD – standard deviation; TX – percent time spent below X% oxygen saturation.*

**Table S7. Comparison of oximetry patterns in adjusted Cox regression analyses for incident Cardiovascular Disease**. Analysis is for the entire sample (N=2863) and oximetry patterns are measured separately within NREM and REM sleep (oximetry patterns for total sleep time are shown for comparison). This table shows the hazard ratios (95% confidence interval) which are displayed in **Figure S2** and includes the exact p values for each association. Models are adjusted for age, body mass index, race, sex, and smoking status.

|  | **Total Sleep** | | **NREM Sleep** | | **REM Sleep** | |
| --- | --- | --- | --- | --- | --- | --- |
| **Parameters by type** | **Hazard ratio (95% CI)** | **P value** | **Hazard ratio (95% CI)** | **P value** | **Hazard ratio (95% CI)** | **P value** |
| *Respiratory Events* |  |  |  |  |  |  |
| **AHI** (events/hr) | 0.99  (0.90, 1.09) | 0.865 | 0.99  (0.90, 1.08) | 0.787 | 1.03  (0.94, 1.14) | 0.526 |
| *Desaturation* |  |  |  |  |  |  |
| **ODI2** (events/hr) | 0.97  (0.88, 1.07) | 0.553 | 0.97  (0.88, 1.06) | 0.507 | 1.03  (0.94, 1.14) | 0.543 |
| **ODI3** (events/hr) | 1.06  (0.88, 1.06) | 0.452 | 0.96  (0.88. 1.06) | 0.418 | 1.03  (0.93, 1.13) | 0.601 |
| **ODI4** (events/hr) | 0.95  (0.87, 1.05) | 0.316 | 0.95  (0.87, 1.05) | 0.328 | 0.99  (0.90, 1.09) | 0.820 |
| **ODI5** (events/hr) | 0.94  (0.85, 1.04) | 0.217 | 0.95  (0.86, 1.04) | 0.266 | 0.96  (0.67, 1.06) | 0.376 |
| **Hypoxic Burden** (%min/hr) | 0.95  (0.86, 1.05) | 0.311 | 0.94  (0.86, 1.05) | 0.311 | 0.94  (0.85. 1.04) | 0.248 |
| *Time series analysis* |  |  |  |  |  |  |
| Frequency distribution |  |  |  |  |  |  |
| **Mean** (%SpO_2_) | 1.01  (0.92, 1.12) | 0.797 | 1.0  (0.91, 1.11) | 0.878 | 1.02  (0.93, 1.14) | 0.640 |
| **SD** (%SpO_2_) | 0.96  (0.86, 1.06) | 0.382 | 0.97  (0.88, 1.07) | 0.541 | 0.95  (0.86, 1.05) | 0.343 |
| **Skewness** (%SpO_2_) | 1.04  (0.95, 1.14) | 0.373 | 1.03  (0.94, 1.12) | 0.574 | 1.03  (0.93, 1.13) | 0.602 |
| **Kurtosis** (%SpO_2_) | 0.95  (0.84, 1.07) | 0.376 | 0.96  (0.87, 1.07) | 0.485 | 0.96  (0.86, 1.08) | 0.493 |
| Cumulative frequency distribution |  |  |  |  |  |  |
| **Median** (%SpO_2_) | 1.00  (0.91, 1.11) | 0.969 | 1.01  (0.91, 1.11) | 0.919 | 1.00  (0.90, 1.10) | 0.981 |
| **Nadir** (%SpO_2_) | 1.08  (0.98, 1.20) | 0.103 | 1.05  (0.96, 1.16) | 0.275 | 1.08  (0.98, 1.20) | 0.111 |
| **T80** (%TST) | 0.82  (0.62, 1.09) | 0.182 | 0.24  (0.05, 1.10) | 0.07 | 0.92  (0.80, 1.05) | 0.216 |
| **T82** (%TST) | 0.88  (0.71, 1.20) | 0.255 | 0.526  (0.25, 1.11) | 0.092 | 0.95  (0.84, 1.07) | 0.368 |
| **T84** (%TST) | 0.92  (0.77, 1.09) | 0.315 | 0.72  (0.49, 1.07) | 0.106 | 0.98  (0.88, 1.08) | 0.654 |
| **T86** (%TST) | 0.96  (0.84, 1.09) | 0.510 | 0.92  (0.77, 1.09) | 0.325 | 1.00  (0.91, 1.10) | 0.993 |
| **T88** (%TST) | 1.00  (0.90, 1.10) | 0.968 | 1.00  (0.90, 1.10) | 0.958 | 1.00  (0.91, 1.10) | 0.971 |
| **T90** (%TST) | 1.05  (0.98, 1.14) | 0.176 | 1.06  (0.99, 1.14) | 0.09 | 1.01  (0.92, 1.10) | 0.840 |
| **T92** (%TST) | 1.07  (0.98, 1.16) | 0.122 | 1.08  (1.0, 1.17) | 0.067 | 1.01  (0.92, 1.10) | 0.837 |
| **T94** (%TST) | 1.01  (0.92, 1.10) | 0.890 | 1.01  (0.92, 1.11) | 0.830 | 1.01  (0.91, 1.11) | 0.897 |
| *Power spectral density* |  |  |  |  |  |  |
| Full frequency band |  |  |  |  |  |  |
| **Mean** ([SpO_2_]^2^) | 0.90  (0.80, 1.02) | 0.111 | 0.92  (0.81, 1.04) | 0.183 | 0.91  (0.81, 1.02) | 0.108 |
| **SD** ([%SpO_2_]^2^) | 0.93  (0.83, 1.04) | 0.189 | 0.95  (0.85, 1.06) | 0.361 | 0.92  (0.83, 1.03) | 0.144 |
| **Skewness** ([%SpO_2_]^2^) | 1.09  (1.0, 1.19) | 0.051 | 1.07  (0.97, 1.16) | 0.191 | 1.03  (0.94, 1.13) | 0.494 |
| **Kurtosis** ([%SpO_2_]^2^) | 1.09  (1.0, 1.18) | 0.053 | 1.06  (0.97, 1.16) | 0.223 | 1.04  (0.95, 1.13) | 0.416 |
| **Spectral entropy** (no units) | 0.98  (0.90, 1.08) | 0.735 | 0.97  (0.89, 1.06) | 0.498 | 1.02  (0.93, 1.12) | 0.659 |
| OSA frequency band |  |  |  |  |  |  |
| **Mean** ([%SpO_2_]^2^) | 0.91  (0.80, 1.03) | 0.126 | 0.92  (0.81, 1.04) | 0.195 | 0.91  (0.82, 1.02) | 0.121 |
| **SD** ([%SpO_2_]^2^) | 0.94  (0.85, 1.05) | 0.282 | 0.96  (0.87, 1.07) | 0.474 | 0.93  (0.83, 1.03) | 0.171 |
| **Skewness** ([%SpO_2_]^2^) | 1.02  (0.94, 1.11) | 0.563 | 1.02  (0.94, 1.11) | 0.577 | 1.00  (0.92, 1.09) | 0.985 |
| **Kurtosis** ([%SpO_2_]^2^) | 1.04  (0.97, 1.11) | 0.324 | 1.01  (0.94, 1.09) | 0.762 | 1.02  (0.93, 1.11) | 0.721 |
| **Spectral entropy** (no units) | 1.00  (0.92, 1.09) | 0.982 | 0.96  (0.88, 1.04) | 0.334 | 1.06  (0.97, 1.16) | 0.228 |
| *Non-linear* |  |  |  |  |  |  |
| **Sample entropy** (no units) | 0.99  (0.89, 1.09) | 0.843 | 0.98  (0.89, 1.08) | 0.691 | 1.07  (0.97, 1.18) | 0.153 |
| **Central tendency measure** (no units) | 1.04  (0.94, 1.15) | 0.431 | 1.06  (0.96, 1.17) | 0.219 | 0.96  (0.87, 1.06) | 0.470 |

**
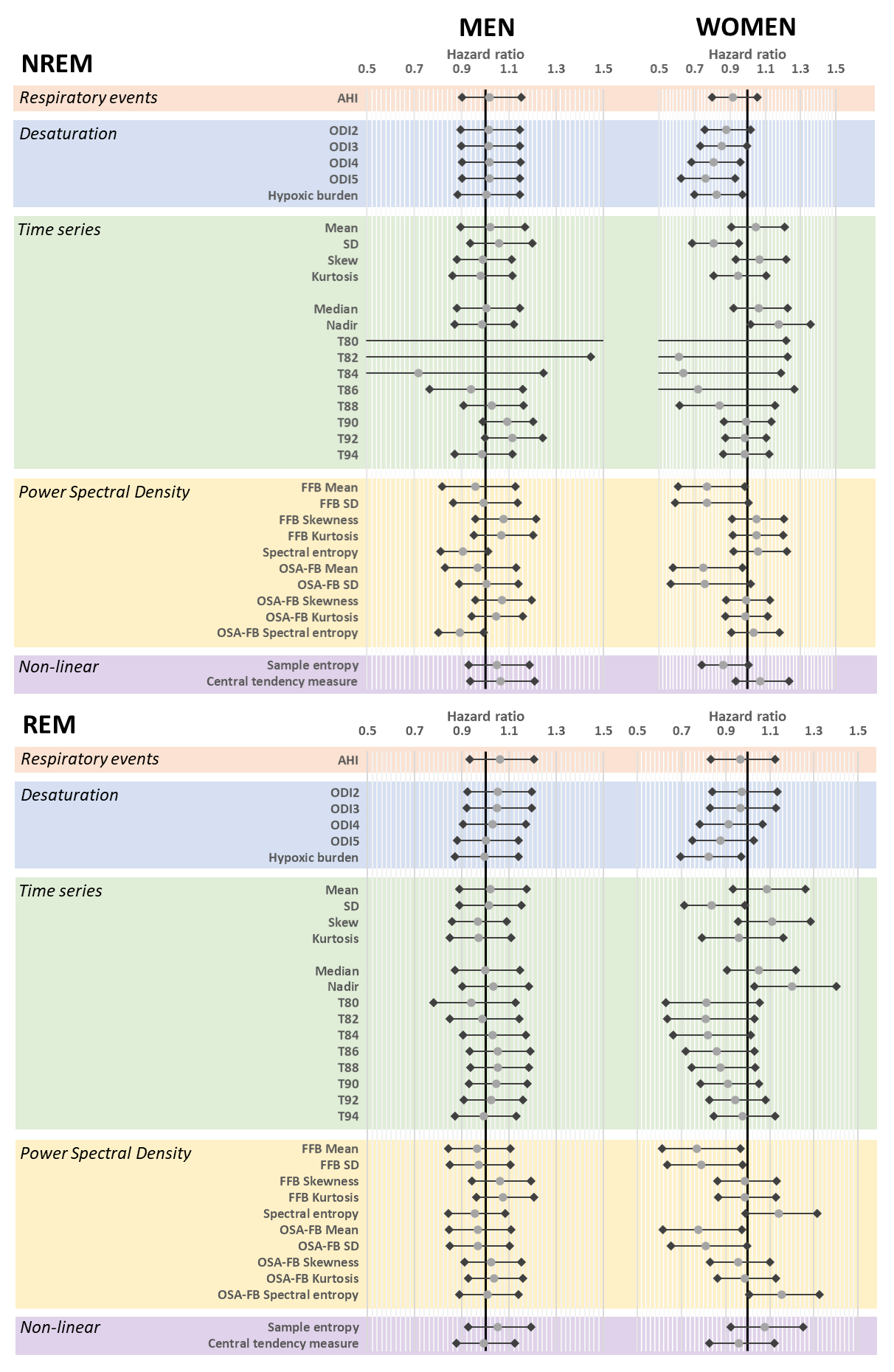
**

**Figure S3. Comparison of oximetry patterns in adjusted Cox regression analyses for incident Cardiovascular Disease in men and women**. Analysis is performed separately for the male sample (n=1380) and female sample (n=1483). Additionally, oximetry patterns are presented separately for NREM (top panel) and REM (bottom panel) sleep. Respiratory events measured by the standard clinical measures of OSA severity, Apnoea Hypopnea Index (AHI), is shown for comparison. Oximetry patterns are derived using four analysis approaches: desaturation characteristics, time series analysis, power spectral density, non-linear analysis. Models are adjusted for age, body mass index, race, sex, smoking status, and AHI. Grey circles represent hazard ratios with black diamonds the 95% confidence interval for the hazard ratio.

*FFB – full frequency band; ODI – oxygen desaturation index; OSA-FB – Obstructive Sleep Apnoea Frequency Band; SD – standard deviation; TX – percent time spent below X% oxygen saturation.*

**Table S8. Comparison of oximetry patterns in adjusted Cox regression analyses for incident Cardiovascular Disease**. Analysis is performed separately for the male sample (n=1380) and female sample (n=1483). Oximetry patterns are presented for NREM sleep only (REM sleep values can be found in **Table S9**). This table shows the hazard ratios (95% confidence interval) which are displayed in **Figure S3** and includes the exact p values for each association. Models are adjusted for age, body mass index, race, sex, and smoking status.

| **NREM SLEEP** | **Men** | | **Women** | |
| --- | --- | --- | --- | --- |
| **Parameters by type** | **Hazard ratio (95% CI)** | **P value** | **Hazard ratio (95% CI)** | **P value** |
| *Respiratory Events* |  |  |  |  |
| **AHI** (events/hr) | 1.02  (0.90, 1.15) | 0.754 | 0.92  (0.80, 1.07) | 0.236 |
| *Desaturation* |  |  |  |  |
| **ODI2** (events/hr) | 1.01  (0.90, 1.15) | 0.813 | 0.88  (0.76, 1.02) | 0.084 |
| **ODI3** (events/hr) | 1.02  (0.90, 1.15) | 0.811 | 0.86  (0.73, 1.0) | 0.048 |
| **ODI4** (events/hr) | 1.02  (0.90, 1.15) | 0.749 | 0.81  (0.68, 0.96) | 0.015 |
| **ODI5** (events/hr) | 1.02  (0.90, 1.15) | 0.770 | 0.76  (0.62, 0.93) | 0.007 |
| **Hypoxic Burden** (%min/hr) | 1.01  (0.88, 1.15) | 0.913 | 0.83  (0.70, 0.97) | 0.023 |
| *Time series analysis* |  |  |  |  |
| Frequency distribution |  |  |  |  |
| **Mean** (%SpO_2_) | 1.02  (0.90, 1.17) | 0.736 | 1.05  (0.91, 1.21) | 0.510 |
| **SD** (%SpO_2_) | 1.06  (0.94, 1.20) | 0.353 | 0.81  (0.67, 0.95) | 0.011 |
| **Skewness** (%SpO_2_) | 0.99  (0.88, 1.11) | 0.863 | 1.07  (0.94, 1.22) | 0.325 |
| **Kurtosis** (%SpO_2_) | 0.98  (0.86, 1.12) | 0.772 | 0.95  (0.81, 1.11) | 0.485 |
| Cumulative frequency distribution |  |  |  |  |
| **Median** (%SpO_2_) | 1.01  (0.88, 1.15) | 0.932 | 1.07  (0.92, 1.23) | 0.386 |
| **Nadir** (%SpO_2_) | 0.99  (0.87, 1.12) | 0.861 | 1.18  (1.02, 1.36) | 0.026 |
| **T80** (%TST) | 0.16  (0.01, 2.01) | 0.156 | 00.50  (0.20, 1.22) | 0.127 |
| **T82** (%TST) | 0.46  (0.15. 1.45) | 0.184 | 0.61  (0.30, 1.23) | 0.167 |
| **T84** (%TST) | 0.72  (0.41, 1.25) | 0.239 | 0.63  (0.34, 1.19) | 0.157 |
| **T86** (%TST) | 0.94  (0.77, 1.16) | 0.567 | 0.72  (0.41, 1.27) | 0.256 |
| **T88** (%TST) | 1.03  (0.91, 1.16) | 0.656 | 0.84  (0.62, 1.16) | 0.288 |
| **T90** (%TST) | 1.09  (0.99, 1.20) | 0.07 | 0.99  (0.87, 1.4) | 0.901 |
| **T92** (%TST) | 1.11  (1.0, 1.25) | 0.052 | 0.98  (0.86, 1.11) | 0.798 |
| **T94** (%TST) | 0.99  (0.87, 1.12) | 0.828 | 0.99  (0.86, 1.12) | 0.831 |
| *Power spectral density* |  |  |  |  |
| Full frequency band |  |  |  |  |
| **Mean** ([SpO_2_]^2^) | 0.96  (0.82, 1.13) | 0.623 | 0.77  (0.61, 0.98) | 0.036 |
| **SD** ([%SpO_2_]^2^) | 0.99  (0.87, 1.14) | 0.915 | 0.77  (0.59, 1.00) | 0.053 |
| **Skewness** ([%SpO_2_]^2^) | 1.08  (0.96, 1.21) | 0.204 | 1.05  (0.92, 1.21) | 0.481 |
| **Kurtosis** ([%SpO_2_]^2^) | 1.07  (0.95, 1.20) | 0.260 | 1.05  (0.92, 1.20) | 0.485 |
| **Spectral entropy** (no units) | 0.91  (0.81, 1.01) | 0.077 | 1.06  (0.92, 1.22) | 0.400 |
| OSA frequency band |  |  |  |  |
| **Mean** ([%SpO_2_]^2^) | 0.97  (0.83, 1.13) | 0.689 | 0.75  (0.58, 0.97) | 0.030 |
| **SD** ([%SpO_2_]^2^) | 1.01  (0.89, 1.34) | 0.919 | 0.76  (0.57, 1.02) | 0.067 |
| **Skewness** ([%SpO_2_]^2^) | 1.07  (0.96, 1.20) | 0.222 | 0.99  (0.88, 1.13) | 0.935 |
| **Kurtosis** ([%SpO_2_]^2^) | 1.05  (0.95, 1.16) | 0.382 | 0.99  (0.88, 1.12) | 0.863 |
| **Spectral entropy** (no units) | 0.89  (0.80, 0.99) | 0.040 | 1.04  (0.91, 1.18) | 0.594 |
| *Non-linear* |  |  |  |  |
| **Sample entropy** (no units) | 1.05  (0.93, 1.19) | 0.423 | 0.86  (0.74, 1.00) | 0.056 |
| **Central tendency measure** (no units) | 1.07  (0.94, 1.21) | 0.324 | 1.07  (0.93, 1.23) | 0.320 |

**Table S9. Comparison of oximetry patterns in adjusted Cox regression analyses for incident Cardiovascular Disease**. Analysis is performed separately for the male sample (n=1380) and female sample (n=1483). Oximetry patterns are presented for REM sleep only (REM sleep values can be found in **Table S8**). This table shows the hazard ratios (95% confidence interval) which are displayed in **Figure S3** and includes the exact p values for each association. Models are adjusted for age, body mass index, race, sex, and smoking status.

| **REM SLEEP** | **Men** | | **Women** | |
| --- | --- | --- | --- | --- |
| **Parameters by type** | **Hazard ratio (95% CI)** | **P value** | **Hazard ratio (95% CI)** | **P value** |
| *Respiratory Events* |  |  |  |  |
| **AHI** (events/hr) | 1.06  (0.93, 1.21) | 0.362 | 0.97  (0.84, 1.12) | 0.677 |
| *Desaturation* |  |  |  |  |
| **ODI2** (events/hr) | 1.05  (0.93, 1.20) | 0.437 | 0.98  (0.84, 1.14) | 0.754 |
| **ODI3** (events/hr) | 1.05  (0.92, 1.20) | 0.451 | 0.97  (0.83, 1.13) | 0.684 |
| **ODI4** (events/hr) | 1.03  (0.91, 1.17) | 0.657 | 0.92  (0.78, 1.07) | 0.265 |
| **ODI5** (events/hr) | 1.00  (0.88, 1.14) | 0.971 | 0.88  (0.75, 1.03) | 0.109 |
| **Hypoxic Burden** (%min/hr) | 1.00  (0.87, 1.14) | 0.960 | 0.82  (0.70, 0.97) | 0.022 |
| *Time series analysis* |  |  |  |  |
| Frequency distribution |  |  |  |  |
| **Mean** (%SpO_2_) | 1.02  (0.89, 1.17) | 0.763 | 1.09  (0.94, 1.26) | 0.277 |
| **SD** (%SpO_2_) | 1.01  (0.89, 1.15) | 0.839 | 0.84  (0.71, 0.99) | 0.034 |
| **Skewness** (%SpO_2_) | 0.97  (0.86, 1.09) | 0.581 | 1.11  (0.96, 1.29) | 0.166 |
| **Kurtosis** (%SpO_2_) | 0.97  (0.85, 1.11) | 0.650 | 0.96  (0.79, 1.16) | 0.685 |
| Cumulative frequency distribution |  |  |  |  |
| **Median** (%SpO_2_) | 1.00  (0.87, 1.15) | 0.995 | 1.05  (0.91, 1.22) | 0.497 |
| **Nadir** (%SpO_2_) | 1.03  (0.90, 1.18) | 0.635 | 1.20  (1.03, 1.40) | 0.018 |
| **T80** (%TST) | 0.94  (0.78, 1.13) | 0.501 | 0.82  (0.63, 1.05) | 0.120 |
| **T82** (%TST) | 0.99  (0.85, 1.14) | 0.846 | 0.81  (0.64, 1.03) | 0.087 |
| **T84** (%TST) | 1.03  (0.90, 1.17) | 0.667 | 0.82  (0.66, 1.01) | 0.067 |
| **T86** (%TST) | 1.05  (0.93,1.19) | 0.408 | 0.86  (0.72, 1.03) | 0.104 |
| **T88** (%TST) | 1.05  (0.94, 1.18) | 0.397 | 0.88  (0.75, 1.03) | 0.12 |
| **T90** (%TST) | 1.05  (0.93, 1.18) | 0.444 | 0.91  (0.79, 1.05) | 0.198 |
| **T92** (%TST) | 1.03  (0.91, 1.16) | 0.690 | 0.95  (0.83, 1.08) | 0.410 |
| **T94** (%TST) | 0.99  (0.87, 1.13) | 0.918 | 0.98  (0.85, 1.12) | 0.739 |
| *Power spectral density* |  |  |  |  |
| Full frequency band |  |  |  |  |
| **Mean** ([SpO_2_]^2^) | 0.97  (0.84, 1.11) | 0.615 | 0.77  (0.61, 0.97) | 0.025 |
| **SD** ([%SpO_2_]^2^) | 0.97  (0.85, 1.11) | 0.644 | 0.79  (0.64, 0.98) | 0.030 |
| **Skewness** ([%SpO_2_]^2^) | 1.06  (0.94, 1.19) | 0.320 | 0.99  (0.86, 1.13) | 0.868 |
| **Kurtosis** ([%SpO_2_]^2^) | 1.08  (0.96, 1.20) | 0.209 | 0.99  (0.87, 1.13) | 0.860 |
| **Spectral entropy** (no units) | 0.96  (0.84, 1.08) | 0.479 | 1.14  (0.99, 1.32) | 0.067 |
| OSA frequency band |  |  |  |  |
| **Mean** ([%SpO_2_]^2^) | 0.97  (0.85, 1.11) | 0.635 | 0.78  (0.62, 0.98) | 0.030 |
| **SD** ([%SpO_2_]^2^) | 0.97  (0.85, 1.10) | 0.618 | 0.81  (0.65, 1.00) | 0.049 |
| **Skewness** ([%SpO_2_]^2^) | 1.03  (0.91, 1.15) | 0.676 | 0.96  (0.83, 1.10) | 0.537 |
| **Kurtosis** ([%SpO_2_]^2^) | 1.04  (0.93, 1.16) | 0.5437 | 0.99  (0.86, 1.13) | 0.841 |
| **Spectral entropy** (no units) | 1.01  (0.89, 1.14) | 0.908 | 1.16  (1.01, 1.33) | 0.040 |
| *Non-linear* |  |  |  |  |
| **Sample entropy** (no units) | 1.05  (0.93, 1.19) | 0.434 | 1.08  (0.93, 1.25) | 0.339 |
| **Central tendency measure** (no units) | 0.99  (0.88, 1.12) | 0.912 | 0.96  (0.83, 1.12) | 0.625 |

**REFERENCES**

1. Quan SF, Howard BV, Iber C, Kiley JP, Nieto FJ, O'Connor GT, Rapoport DM, Redline S, Robbins J, Samet JM, Wahl PW. The Sleep Heart Health Study: design, rationale, and methods. *Sleep* 1997: 20(12): 1077-1085.

2. Redline S, Sanders MH, Lind BK, Quan SF, Iber C, Gottlieb DJ, Bonekat WH, Rapoport DM, Smith PL, Kiley JP. Methods for obtaining and analyzing unattended polysomnography data for a multicenter study. Sleep Heart Health Research Group. *Sleep* 1998: 21(7): 759-767.

3. Redline S, Yenokyan G, Gottlieb DJ, Shahar E, O'Connor GT, Resnick HE, Diener-West M, Sanders MH, Wolf PA, Geraghty EM, Ali T, Lebowitz M, Punjabi NM. Obstructive sleep apnea-hypopnea and incident stroke: the sleep heart health study. *Am J Respir Crit Care Med* 2010: 182(2): 269-277.

4. Berry RB, Budhiraja R, Gottlieb DJ, Gozal D, Iber C, Kapur VK, Marcus CL, Mehra R, Parthasarathy S, Quan SF, Redline S, Strohl KP, Davidson Ward SL, Tangredi MM, American Academy of Sleep M. Rules for scoring respiratory events in sleep: update of the 2007 AASM Manual for the Scoring of Sleep and Associated Events. Deliberations of the Sleep Apnea Definitions Task Force of the American Academy of Sleep Medicine. *J Clin Sleep Med* 2012: 8(5): 597-619.

5. Rechtschaffen A. A manual for standardized terminology, techniques and scoring system for sleep stages in human subjects. *Brain information service* 1968.
